# Systems biology of interstitial lung diseases: integration of mRNA and microRNA expression changes

**DOI:** 10.1186/1755-8794-4-8

**Published:** 2011-01-17

**Authors:** Ji-Hoon Cho, Richard Gelinas, Kai Wang, Alton Etheridge, Melissa G Piper, Kara Batte, Duaa Dakhallah, Jennifer Price, Dan Bornman, Shile Zhang, Clay Marsh, David Galas

**Affiliations:** 1 Institute for Systems Biology, Seattle WA USA; 2 Division of Health and Life Sciences, Battelle Memorial Institute, Columbus OH USA; 3 Division of Pulmonary, Allergy, Critical Care, Sleep Medicine, Davis Heart and Lung Research Institute, Columbus, OH USA; 4 University of Luxembourg, 7220 Walfer, Luxembourg

## Abstract

**Background:**

The molecular pathways involved in the interstitial lung diseases (ILDs) are poorly understood. Systems biology approaches, with global expression data sets, were used to identify perturbed gene networks, to gain some understanding of the underlying mechanisms, and to develop specific hypotheses relevant to these chronic lung diseases.

**Methods:**

Lung tissue samples from patients with different types of ILD were obtained from the Lung Tissue Research Consortium and total cell RNA was isolated. Global mRNA and microRNA were profiled by hybridization and amplification-based methods. Differentially expressed genes were compiled and used to identify critical signaling pathways and potential biomarkers. Modules of genes were identified that formed a regulatory network, and studies were performed on cultured cells *in vitro *for comparison with the *in vivo *results.

**Results:**

By profiling mRNA and microRNA (miRNA) expression levels, we found subsets of differentially expressed genes that distinguished patients with ILDs from controls and that correlated with different disease stages and subtypes of ILDs. Network analysis, based on pathway databases, revealed several disease-associated gene modules, involving genes from the TGF-β, Wnt, focal adhesion, and smooth muscle actin pathways that are implicated in advancing fibrosis, a critical pathological process in ILDs. A more comprehensive approach was also adapted to construct a putative global gene regulatory network based on the perturbation of key regulatory elements, transcription factors and microRNAs. Our data underscores the importance of TGF-β signaling and the persistence of smooth muscle actin-containing fibroblasts in these diseases. We present evidence that, downstream of TGF-β signaling, microRNAs of the miR-23a cluster and the transcription factor Zeb1 could have roles in mediating an epithelial to mesenchymal transition (EMT) and the resultant persistence of mesenchymal cells in these diseases.

**Conclusions:**

We present a comprehensive overview of the molecular networks perturbed in ILDs, discuss several potential key molecular regulatory circuits, and identify microRNA species that may play central roles in facilitating the progression of ILDs. These findings advance our understanding of these diseases at the molecular level, provide new molecular signatures in defining the specific characteristics of the diseases, suggest new hypotheses, and reveal new potential targets for therapeutic intervention.

## Background

The interstitial lung diseases (ILDs), a broad category of restrictive lung disorders, exhibit cellular infiltration and distortion of the interstitium and alveolar gas units [1]. Current descriptions of ILDs at the tissue or cell levels include broadly defined processes such as aberrant wound repair, scarring, apoptosis, or fibrosis, whereas at the molecular level, these diseases are associated with dysregulation of a complex set of cytokines, growth factors, and signaling molecules [1-4]. In particular, the TGF-β [4-6] and the Wnt signaling pathways [4,7,8] are thought to have key roles in the disease. Recently, through global gene expression profiling, several studies have revealed more fundamental processes involved ILDs, including extracellular matrix remodeling [9], alterations in the cytoskeleton [10], and the possibility that a process similar to the developmental epithelial to mesenchymal cell transition gives rise to the fibroblasts that are prominent in idiopathic pulmonary fibrosis and the other ILDs [6,10]. In addition to understanding the disease better, gene expression profiling results have also improved the pathological classification of the ILDs [11,12].

MicroRNAs (miRNAs), small, 21-25 nucleotide long non-coding RNAs, can regulate global gene networks by interacting with specific messenger RNAs (mRNAs) to repress translation or hasten mRNA degradation [13,14]. Based on a computational prediction model, microRNA molecules may also be hubs in the regulation of gene networks, as a single miRNA can affect the function of numerous mRNAs [15]. In fact, as many as one-third of all mRNAs, including a large number of transcription factors may be regulated by miRNAs [16,17]. Specific miRNAs have already been implicated in lung biology [18-20]; for example, deleting miRNAs of the miR-17~miR-92 cluster prevents normal lung development [21], while over-expression of this cluster leads to epithelial cell proliferation in the lung [18].

The systems biology approach is to view the biological system as a whole in order to study the effects of disease and global interactions with the environment, which facilitates understanding of biological processes and disease [22,23]. The five key components for systems biology are the global measurements of biomolecules, the integration of biological information, the identification of molecular responses to perturbations, the building of testable models, and the refinement of models through testing of these hypotheses. Once the models accurately represent the specific biological responses, they can be used to understand disease progression, identify new disease intervention points, and predict the molecular responses to perturbations [24].

To better understand the complex pathology associated with ILDs and identify molecular networks involved in the disease, we took a systems biology approach to acquire and integrate changes of mRNA and miRNA levels between biopsy samples from patients with ILD and from normal lung tissue. First, we found distinct molecular signatures that distinguish patients from controls, and that may differentiate patients with one subtype of ILD (IPF, idiopathic pulmonary fibrosis) from another type (NSIP, non-specific interstitial pneumonitis). Next, we identified known signaling pathways that were characterized with differentially expressed modules of genes in greater detail than in previous studies. We then integrated differentially expressed mRNAs with selected, differentially expressed miRNAs and transcription factors (TFs) and produced an initial global view of the molecular changes that are present in the ILDs. This analysis also revealed several potential disease-associated regulatory circuits, including events downstream of TGF-β signaling that may play a role in disease persistence. We present data showing that miRNAs in the miR-23a cluster, downstream of TGF-β and Zeb-1, may have a role as positive regulators of the epithelial to mesenchymal transition, which has been proposed to play an important role in ILD pathogenesis. While there are aspects of the ILDs that our models do not describe, by using a systems approach we have identified several potentially important regulatory networks, leading to new candidates for biomarkers for disease stratification and progression, and putative therapeutic targets for these debilitating diseases.

## Methods

### Ethics Statement

The project was conducted in compliance with U.S. 45 CFR 46 ("Common Rule") as administered by the Office of Human Research Protections within the United States Department of Health and Human Services. The Common Rule is derived from the basic ethical principles espoused in the Belmont Report (http://ohsr.od.nih.gov/guidelines/belmont.html). This project utilized de-identified tissue samples acquired through the Lung Tissue Research Consortium (LTRC #07-99-0006; ltrcpublic.com), Lifeline of Ohio and the Cooperative Human Tissue Network (CHTN). The use of these samples was approved at the Ohio State University under IRB protocol 2007H0002.

### Description of study population

Lung tissue samples from thirty patients with IPF or related disorders were obtained from the Lung Tissue Research Consortium (http://www.ltrcpublic.com). Ten samples came from patients who had been diagnosed with usual interstitial pneumonia/idiopathic pulmonary fibrosis (UIP/IPF), nine samples came from patients with non-specific interstitial pneumonia (NSIP), four from patients with uncharacterized fibrosis, and the remaining samples came from patients with other ILD variants (Table 1). Biopsies from uninvolved lung tissue from lung cancer patients (5 samples) and from one lung transplant patient were used as controls for comparison with the IPF samples.

**Table 1:** Demographic and clinical information of 29 samples used for mRNA/miRNA profiling

Sample ID	Group	FVC %	Gender	Age	Race	Cigarette smoking	Clinical diagnosis	RNA profiling
4	1	< 50%	Male	37	Caucasian	Unknown	UIP/IPF	mRNA/miRNA
6	1	< 50%	Female	45	African-American	Never	UIP/IPF	mRNA/miRNA
7	1	< 50%	Female	26	Caucasian	Never	FU	mRNA/miRNA
9	1	< 50%	Male	64	Caucasian	Previous	UIP/IPF	mRNA
10	1	< 50%	Female	58	Caucasian	Never	UIP/IPF	mRNA/miRNA
11	2	50~80%	Male	58	Caucasian	Unknown	UIP/IPF	mRNA/miRNA
12	2	50~80%	Female	66	Caucasian	Never	NSIP	mRNA
13	2	50~80%	Male	57	Caucasian	Previous	NSIP	mRNA/miRNA
14	2	50~80%	Male	60	Caucasian	Unknown	UIP/IPF	mRNA/miRNA
16	2	50~80%	Male	61	Caucasian	Previous	UIP/IPF	mRNA/miRNA
17	2	50~80%	Female	69	Caucasian	Never	HP	mRNA/miRNA
19	2	50~80%	Male	68	Caucasian	Previous	UIP/IPF	mRNA/miRNA
20	2	50~80%	Male	80	Caucasian	Previous	NSIP	mRNA
21	3	> 80%	Male	65	Caucasian	Previous	NSIP	mRNA/miRNA
22	3	> 80%	Male	52	Hispanic	Never	RB-ILD	mRNA/miRNA
23	3	> 80%	Male	39	Caucasian	Previous	RB-ILD	mRNA/miRNA
24	3	> 80%	Male	61	Caucasian	Previous	COP	mRNA/miRNA
25	3	> 80%	Female	68	Caucasian	Never	UIP/IPF	mRNA
26	3	> 80%	Male	53	Caucasian	Never	COP	mRNA/miRNA
27	3	> 80%	Female	50	African-American	Previous	NSIP	mRNA/miRNA
28	3	> 80%	Female	66	Caucasian	Previous	HP	mRNA/miRNA
29	3	> 80%	Male	64	Caucasian	Previous	UIP/IPF	mRNA/miRNA
30	3	> 80%	Male	56	Caucasian	Previous	UIP/IPF	mRNA/miRNA
43	Normal	-	Female	71	Caucasian	Unknown	Uninvolved carcinoma tissue	mRNA/miRNA
44	Normal	-	Female	81	Caucasian	Unknown	Uninvolved carcinoma tissue	mRNA/miRNA
46	Normal	-	Male	50	Caucasian	Unknown	Uninvolved carcinoma tissue	mRNA/miRNA
48	Normal	-	Male	73	Caucasian	Unknown	Uninvolved carcinoma tissue	mRNA/miRNA
50	Normal	-	Female	73	Caucasian	Unknown	Uninvolved carcinoma tissue	mRNA/miRNA
51	Normal	-	Male	29	Unknown	Previous	Lung transplant patient	mRNA/miRNA

**Abbreviation **: FVC (forced vital capacity), UIP/IPF (usual interstitial pneumonitis/idiopathic pulmonary fibrosis), NSIP (non-specific interstitial pneumonitis), HP (hypersensitive pneumonitis), COP (cryptogenic organizing pneumonia), RB-ILD (respiratory bronchiolitis-interstitial lung disease)

### RNA isolation and microarray profiling

Total RNA was isolated from individual human lung samples by homogenization in TRIzol^® ^Reagent per the manufacturer's protocol (Invitrogen, Carlsbad, CA) using Soft Tissue Omni Homogenizer Tips (Omni International; Marietta, GA). The quality and quantity of the RNA samples were evaluated for concentration and purity using a NanoDrop ND-1000 Spectrophotometer (NanoDrop; Wilmington, DE), and for integrity by gel electrophoresis using the FlashGel^® ^RNA cassette system (Lonza; Rockland, ME). The mRNA expression profiles in the samples were obtained with Affymetrix GeneChip^® ^Human Genome U133 Plus 2.0 Array (containing 39,000 genes) as described in the Affymetrix GeneChip^® ^protocol. MicroRNAs were profiled by using microarrays from Agilent Technologies (G4470B, Santa Clara, CA) as well as by RT-PCR. The levels of selected mRNA and miRNA observed on microarray studies were validated by using a SYBR-based quantitative RT-PCR method [25]. More detailed protocols are in Additional file 1.

### Microarray data analysis and network construction

Affymetrix array data was summarized, normalized, and transformed in log_2_-scale by the GCRMA method [26]. For the Agilent miRNA array, a consolidated intensity value for each probe was obtained, quantile normalized [27] and log_2_-transformed. Probe sets were identified as present or absent by applying a Gaussian mixture model. We used only these present mRNA probe sets for subsequent statistical analysis, but for miRNA data, all probes were used. A detailed description of data analysis and network construction is given in Additional file 1. All microarray datasets have been uploaded to the GEO database. mRNA results are in GSE21369 while miRNA results are in GSE21394. A 'super series' designation of GSE21411 was assigned to both of these datasets.

### MDCK cell culture

MDCK cells (ATCC, Manassas, VA) were maintained in DMEM containing 10% fetal bovine serum, 2 mM L-glutamine, 0.1 mM non-essential amino acids, 100 U of penicillin, and 100 μg streptomycin (Invitrogen). For isolation of epithelial and mesenchymal subclones, single cells were sorted into 96-well plates using a FACSAria II cell sorter (BD Biosciences, San Jose, CA). Clones were picked based on morphology. Clones with clear epithelial or mesenchymal morphology were expanded. Canine Zeb1 cDNA was amplified by PCR and cloned into pcDNA3.1 directional TOPO expression vector (Invitrogen). Cells were transfected using Lipofectamine 2000 (Invitrogen) according to the manufacturer's protocol. Zeb1-transfected cells were selected with G418 at 1 mg/mL for approximately 3 weeks.

### Western blots

T75 flasks of MDCK cells were rinsed once in PBS and then lysed in NP40 lysis buffer (Invitrogen) containing protease inhibitor cocktail (Pierce, Rockford, IL) for 30 minutes on ice. Lysates were cleared by centrifugation at 13,000 rpm at 4°C for 20 minutes. Lysates were run on a 4-12% NuPAGE gradient gel (Invitrogen) and transferred to Immobilon-FL membrane (Millipore, Billerica, MA). The membrane was blocked for 1 hour in 5% nonfat dry milk diluted in PBS, then incubated overnight at 4°C in primary antibodies diluted in 5% bovine serum albumin in PBS. Primary antibodies were used as follows: rabbit anti-Nedd4L antibody (1:1000; Cell Signaling Technology, Danvers, MA) and mouse anti-Ago2 (1:1000; Abcam, Cambridge, MA). After rinsing 5× in PBS + 0.1% Tween-20 (PBT), the membrane was incubated for 1 hour in donkey anti-mouse 680LT and goat anti-rabbit 800CW secondary antibodies (LI-COR biosciences, Lincoln, NE) in PBS +0.2% Tween-20 and 0.01% SDS. After 5 more rinses in PBT, the membrane was scanned on a LI-COR Odyssey scanner (LI-COR).

### Quantitative PCR

RNA was isolated from MDCK cells using the Qiagen miRNeasy kit according to the manufacturer's protocol (Qiagen, Valencia, CA). For each RNA sample, 150 ng of RNA was reverse transcribed using the miScript kit (Qiagen). cDNAs were diluted 10-fold then used in SYBR-based qPCR reactions. Primers for canine miR-23a, 24, 27a and for canine Zeb1 and Gusb mRNAs were ordered from IDT (Coralville, IA).

## Results

### Sample characteristics

Thirty post-surgical tissue specimens obtained from the Lung Tissue Research Consortium (http://www.ltrcpublic.com) from patients diagnosed with different types of ILDs, and more specific diagnoses, as described in Table 1, were analyzed. To validate the pathology of these samples, histological examinations were conducted on a limited number of randomly picked samples. The results displayed a classical histopathological profile for ILD tissues: replacement of regular alveolar structures with extensive scar tissue and extracellular matrix (Additional file 2).

Ten specimens were obtained in each of three Forced Vital Capacity (FVC) groups, less than 50% (FVC 1), 50 to 80% (FVC 2), and greater than 80% (FVC 3). Due to RNA quality or unsuccessful microarray hybridization in some of the samples, we obtained five mRNA profiling results from the FVC 1 group, eight profiles from the FVC group 2, and ten profiles from the FVC group 3. For controls, one specimen of lung tissue from a normal donor and 11 specimens of uninvolved lung tissue from patients with lung cancer were processed, ostensibly free of interstitial lung disease. Six of these 12 samples gave acceptable global gene expression profiles. We also obtained miRNA expression information for the 6 control and 19 of the 23 ILD samples for which we have mRNA expression information (Table 1).

### Differentially expressed genes and microRNAs

**Figure 1:**
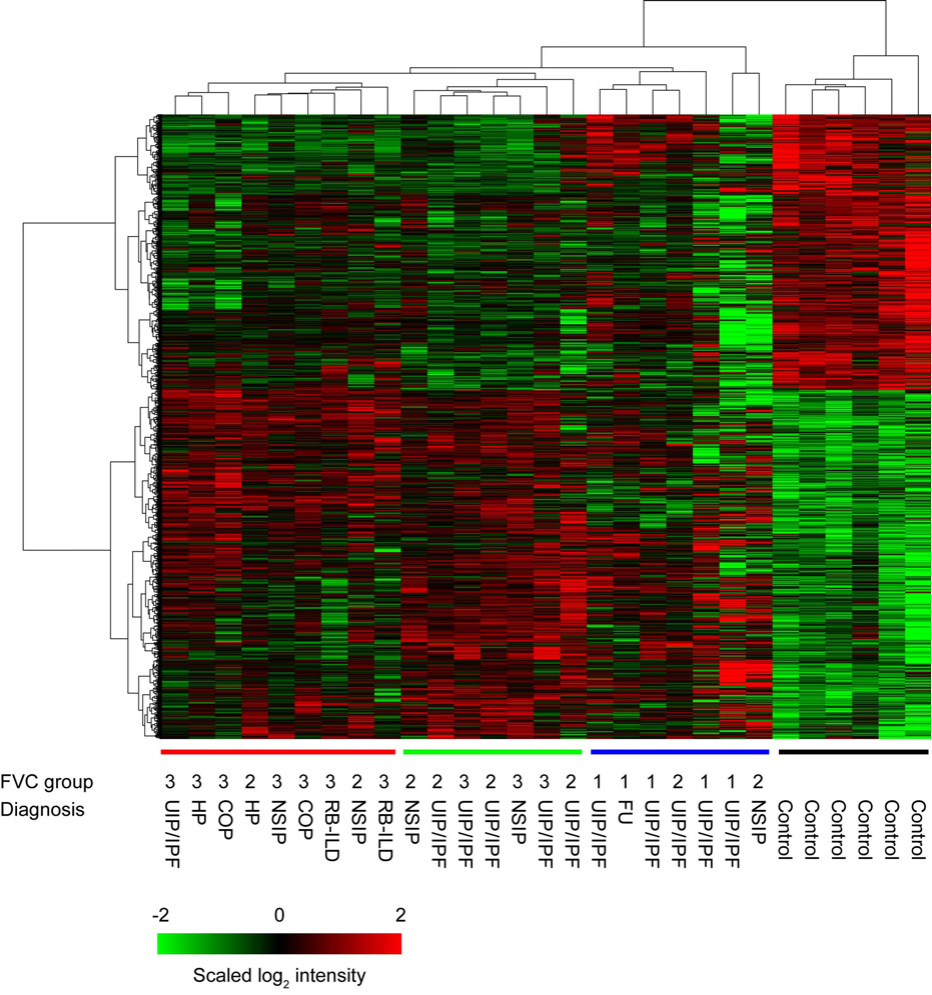
**Hierarchical clustering with 1423 differentially expressed genes between ILD patient and normal control lung samples**. Each row represents the expression profile of a gene across 29 samples and each column represents a sample. The sample IDs and clinical information (FVC group and diagnosis) are listed below the heatmap. Red or green colors indicate either higher or lower expression levels of the gene. Based on the dendrogram, the samples can be further separated into four subgroups indicated by red, green, blue and black bars below the heatmap which correlate with ILD other than UIP/IPF, less severe UIP/IPF (FVC 2 and 3), more severe UIP/IPF (FVC 1), and normal control, respectively.

We focused our initial efforts on identifying genes that were significantly altered in ILD tissues relative to controls by a factor of 1.5-fold or greater, with a positive false discovery rate (pFDR) of < 0.1 (see Additional file 1). Using these criteria, we identified 1423 differentially expressed genes (DEGs), where 795 genes were over-expressed and 628 genes were under-expressed in ILD samples relative to the controls. The changes of selected DEGs were verified with qPCR (Additional file 3). Using hierarchical clustering analysis, the DEGs clustered the samples into two very distinct groups: one group with ILD patient samples and the other with normal controls (Figure 1). In the ILD cluster, the DEGs also separated the samples into three different subgroups (Figure 1), which generally correlated with the disease types and lung function.

**Figure 2:**
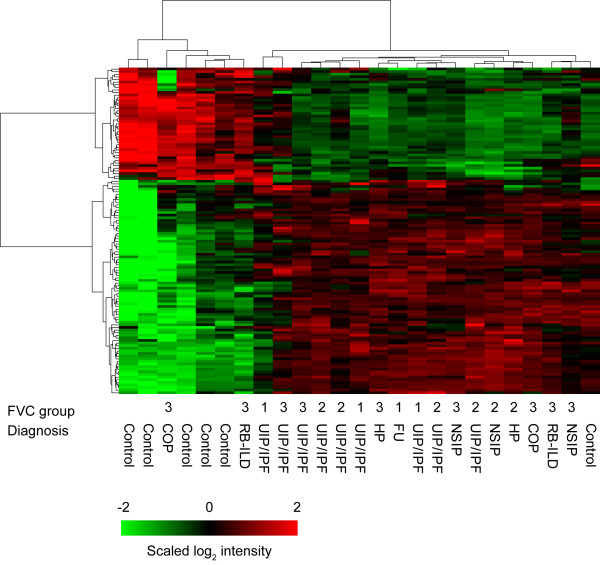
**Hierarchical clustering result of 125 differentially expressed miRNAs**. The sample IDs and clinical information (FVC group and diagnosis) are listed below the heatmap. Red or green colors indicate either higher or lower expression levels of the miRNA. Samples are well separated into control and ILD patient groups except two.

Similarly, we also identified 125 differentially expressed miRNAs (DEmiRNAs) between the ILD patient samples and controls. Among the 125 DEmiRNAs, 82 showed higher levels in the ILD samples compared to the controls. We observed a slight, but interesting, gender bias in the expression levels of DEmiRNAs (lower levels in females), especially for the normal samples (data not shown). Using the same hierarchical clustering approach, the samples can also be grouped into two major classes based only on the DEmiRNAs (Figure 2). While most of the normal samples were separated from the ILDs, one cryptogenic organizing pneumonia (COP) sample was grouped with the controls and one normal sample was grouped with the ILDs. The clustering results from DEmiRNAs did not exhibit a clear separation of the samples either by type or disease severity (FVC groups) as the DEGs did.

### ILDs can be subclassified by using pairs of DEGs and DEmiRNAs

Several pairs of genes and miRNAs that could discriminate samples between ILDs and controls, and among different FVC groups, were identified by ranking pairs of genes and miRNAs that had reciprocal expression profiles between the groups of samples (see Additional file 4) [28]. The most robust pairs of genes and miRNAs, collagen, type III, alpha 1 (*COL3A1*) and Rho guanine nucleotide exchange factor 7 (*ARHGEF7*), and miR-99b and miR-21*, could perfectly discriminate the ILD samples from controls in this set (see Additional file 5).

The same approach was applied to segregate IPF samples from NSIP. NSIP showed distinct clinical outcomes and pathology when compared to IPF [29], but the two disease types displayed extreme similarity in their overall gene and miRNA expression profiles. A few genes, however, showed significant differences between the two disease types. For example, the ratio between hydroxysteroid (17-beta) dehydrogenase 6 homolog (*HSD17B6*) and a hypothetical protein, *C2orf40 *expression levels, separate NSIP and IPF clearly (see Additional file 5).

### Identifying pathways associated with ILD

The lists of DEGs and DEmiRNAs provide an inventory of molecules affected by the diseases. These lists are valuable in the identification of potential biomarkers for the diseases, but to gain a better understanding of the functional implications of these mRNAs and miRNAs, we performed an enrichment analysis to identify KEGG pathways [30] associated with DEGs and to acquire functional correlations from the Gene Ontology (GO) project [31].

The 795 over-expressed DEGs in ILD lungs are strongly associated with pathways including ECM-receptor interaction, cell-cell communication, focal adhesion and B-cell signaling, based on the results of pathway enrichment analyses. Innate immune responses (Toll-like receptor signaling pathways), adipocytokine signaling pathways and metabolic processes are associated with down-regulated DEGs in ILD samples (see Table 2 for a list of some key pathways). The full list of enriched pathways is given in Additional file 1. The Wnt and insulin signaling pathways are associated with a similar number of up- and down-regulated genes which probably reflect the heterogeneity of the tissue samples and dynamic nature of the diseases. Detailed molecular descriptions of the key pathways involved in the ILDs are provided as Additional file 6.

**Table 2:** KEGG pathways enriched by DEGs, all DEmiRNA target genes and all DEmiRNA target DEGs.

		All DEGs		All DEmiRNA predicted targets		DEGs that are DEmiRNA predicted targets	
**KEGG pathway**		**Up-regulated DEGs**	**Down-regulated DEGs**	**All targets of down-regulated DEmiRNAs**	**All targets of up-regulated DEmiRNAs**	**DEG targets of down-regulated DEmiRNAs**	**DEG targets of up-regulated DEmiRNAs**

Signal Transduction	Calcium signaling pathway	4.0E-01	1.0E + 00	**4.6E-04**	**2.7E-03**	4.1E-02	9.5E-01
	ErbB signaling pathway	3.4E-01	3.5E-01	***9.3E-05***	***6.4E-08***	1.3E-01	4.2E-01
	Hedgehog signaling pathway	4.2E-02	4.7E-01	2.8E-02	**6.2E-04**	**8.1E-03**	6.1E-01
	Jak-STAT signaling pathway	6.0E-01	3.9E-01	7.6E-02	7.6E-02	1.0E-01	2.5E-01
	MAPK signaling pathway	2.0E-01	9.5E-02	***1.7E-08***	***1.3E-16***	1.3E-01	1.6E-01
	mTOR signaling pathway	5.3E-01	2.0E-01	1.1E-02	***2.6E-07***	1.0E + 00	5.3E-02
	Notch signaling pathway	7.2E-02	1.6E-01	1.4E-01	**5.4E-03**	2.9E-01	5.4E-01
	Phosphatidylinositol signaling system	2.5E-01	8.6E-01	***8.2E-05***	***3.9E-06***	1.1E-01	3.6E-01
	TGF-beta signaling pathway	3.4E-01	5.5E-01	***4.3E-08***	***1.9E-06***	4.8E-01	1.0E + 00
	VEGF signaling pathway	2.5E-01	1.2E-01	7.4E-02	**2.2E-03**	4.3E-01	3.6E-02
	Wnt signaling pathway	**4.9E-03**	**7.4E-03**	***1.4E-05***	***1.3E-14***	**7.2E-04**	**3.3E-03**

Signaling Molecules and interactions	Cell adhesion molecules (CAMs)	8.1E-02	9.4E-01	8.4E-01	2.9E-01	7.4E-02	6.5E-01
	ECM-receptor interaction	***5.7E-07***	9.0E-01	**2.7E-04**	***9.3E-06***	**3.1E-03**	1.0E + 00

Behavior	Circadian rhythm - mammal	1.0E + 00	1.1E-01	3.1E-02	1.6E-02	1.0E + 00	1.9E-01

Cell Communication	Adherens junction	1.2E-01	9.7E-01	**1.1E-04**	***1.1E-09***	4.3E-01	1.0E + 00
	Focal adhesion	***9.6E-09***	5.5E-01	***1.4E-07***	***8.8E-17***	**3.1E-03**	8.5E-01
	Gap junction	3.7E-01	1.0E + 00	1.1E-02	***4.7E-06***	2.8E-02	1.0E + 00
	Tight junction	4.7E-01	3.9E-01	**8.3E-03**	***3.9E-06***	6.3E-01	1.8E-01

Cell Growth and Death	Apoptosis	8.1E-01	4.4E-02	2.7E-02	**6.5E-03**	1.0E + 00	4.3E-01
	Cell cycle	9.4E-01	3.5E-01	**7.3E-03**	1.4E-01	6.2E-01	8.9E-01
	p53 signaling pathway	6.8E-01	8.6E-02	1.5E-02	**4.4E-03**	4.0E-01	3.1E-01

Cell Motility	Regulation of actin cytoskeleton	1.9E-01	5.0E-01	***2.2E-05***	***1.4E-12***	4.7E-01	4.8E-01

Circulatory System	Vascular smooth muscle contraction	***5.6E-06***	1.0E + 00	**3.0E-03**	**1.0E-03**	**1.6E-04**	1.0E + 00

Development	Axon guidance	6.8E-02	6.9E-01	***1.2E-08***	***3.9E-15***	6.7E-02	3.6E-01
	Dorso-ventral axis formation	5.8E-01	1.0E + 00	**4.0E-04**	1.3E-02	1.0E + 00	1.0E + 00

Endocrine System	Adipocytokine signaling pathway	3.9E-01	**1.5E-04**	2.0E-02	***2.3E-05***	3.9E-01	**7.4E-04**
	GnRH signaling pathway	2.6E-01	4.8E-01	**4.8E-03**	**1.6E-04**	3.8E-02	2.4E-01
	Insulin signaling pathway	**2.2E-03**	1.9E-02	**2.6E-03**	***7.0E-11***	7.9E-02	2.5E-02
	Melanogenesis	6.8E-01	9.9E-01	**2.9E-03**	***1.2E-06***	1.7E-01	8.2E-01
	PPAR signaling pathway	1.0E + 00	1.9E-01	6.6E-01	8.1E-01	1.0E + 00	2.6E-02

With genes in each module of the network, pathway enrichment analysis was performed using one-sided Fisher exact test. The significance level of p-value is represented by regular (0.01~0.1), bold (0.0001~0.01) and bold-italic (<0.0001) fonts.

To explore the possible functional implications of the observed changes in miRNA expression profiles between ILDs and controls, we assembled a comprehensive list of all the potential DEmiRNA target genes identified by TargetScan (version 5.1) [32]. These 7547 presumptive mRNA targets were searched against the master list of differentially expressed mRNAs and 805 were found in common. Pathways enriched by these putative DEmiRNA-targeted DEGs were similar to the pathways associated with the entire 1423 DEGs described earlier. The high degree of consistency in the enriched pathways clearly suggests coherent interactions between the miRNAs and mRNAs and the involvement of these significantly enriched common pathways in the disease (see Table 2 and Additional file 1). Keeping in mind that mRNA levels may not be affected by those miRNAs that regulate translation efficiency, and that the levels of miRNA need not always result in mRNA level changes, this observation on the consistency of affected pathways is highly significant.

**Figure 3:**
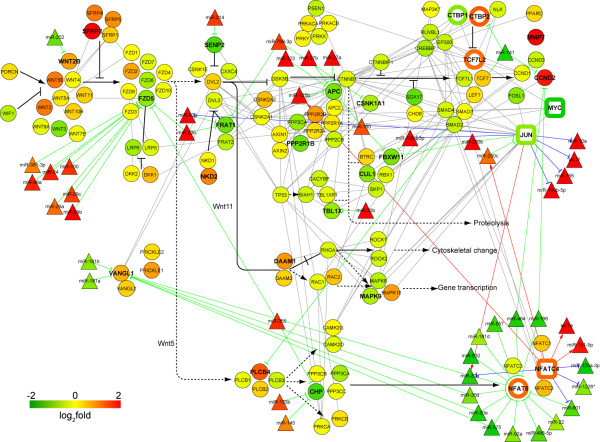
**Integrated view of Wnt pathway**. All (present) genes associated with the Wnt pathway are denoted with by circular nodes and their fold-change values are represented by node color where red and green indicate higher and lower expression in ILD patient samples compared to controls, respectively. Bold-faced gene symbols indicate DEGs. Triangular nodes represent DEmiRNAs which have reciprocal expression fold-change values relative to their presumptive mRNA targets or that are predicted to be regulated by differentially expressed transcription factors (FDR<0.1, see Additional file 1). The transcription factors used in construction of this regulatory network are shown as squares and their fold-changes were expressed by node border colors. Gray and black lines indicate protein-protein interactions and signaling information from the KEGG database, respectively. Red, blue and green lines with arrows represent predicted transcriptional activation, transcriptional repression and miRNA-mediated repression, respectively. Open circles represent transcription factors that were not used in prediction of regulatory interactions.

Using the DEmiRNA-targeted DEGs, we identified 6555 putative DEmiRNA-DEG interaction pairs. Since miRNAs are thought to be largely negative regulators of their targeted genes, the list of DEmiRNA-DEG interactions was reduced to 1530 pairs with 107 distinct DEmiRNAs and 440 DEGs (FDR<0.1, see Additional file 1), by selecting those with inverse correlation on their expression patterns. As previously reported [33], results from our pathway enrichment analyses using DEGs and DEmiRNA targets also indicated a strong involvement of the Wnt pathway in ILDs. The Wnt pathway has been implicated in the control of tissue homeostasis in metazoan organisms, and dysregulation of the pathway, leads to a variety of abnormalities in many tissues, including developmental defects, cancers, and neurological disorders [34]. To elucidate the complex interactions between mRNAs and miRNAs in the Wnt signaling network in ILDs, we examined these presumptive DEmiRNA-DEG interactions in some detail (Figure 3). Several differentially expressed genes between ILD samples and controls in Wnt pathway are heavily targeted by DEmiRNAs; for example, F-box and WD repeat domain containing 11 (*FBXW11*) and frizzled homolog 5 (*FZD5*) (Figure 3, green circles) were down-regulated and likely were "targeted" by up-regulated DEmiRNAs (Figure 3, red triangles) such as miR-199-3p and miR-200b, while the nuclear factor of activated T-cells 5 (*NFAT5*) is up-regulated and targeted by down-regulated DEmiRNAs including miR-30 family members and others (Figure 3, green triangles). The cell surface receptor VanGogh-like 1 (*VANGL1*) is also up-regulated and it is another predicted target of down-regulated miR-30 and miR-181 family members. As discussed later, we suspect that the net effect of this miRNA targeting is to keep signaling through the Wnt pathway persistent and chronic, leading to effective disease progression and persistence.

### The transcription factor and miRNA mediated molecular network presents a systems view of the ILD family of diseases

Besides using manually curated KEGG pathways to assess functional implications of the DEGs and DEmiRNAs, we also took a more global approach by focusing specifically on differentially expressed transcription factors (DETFs) and miRNAs, of which any alteration may lead to profound changes in the normal global molecular network. Among the DEGs, there were 106 transcription factors or putative transcription factors identified [35], where 65 of them showed higher levels in the ILD samples. For example, myocardin (*MYOCD*), a smooth muscle fibroblast-associated transcription factor [36], was one of the most elevated TFs in the ILD samples, while the most suppressed TF in the ILD samples was nuclear receptor subfamily 4, *NR4A2*, a pulmonary epithelial cell differentiation-associated gene [37]. This finding fits well with ILD pathology: a significant increase of myofibroblasts accompanied by a sharp decrease of lung pulmonary epithelial cells. The changes of *MYOCD *and *NR4A2 *mRNA levels in ILD imply the possibility of using TF-mediated networks to gain insight into the state of pathology.

**Figure 4:**
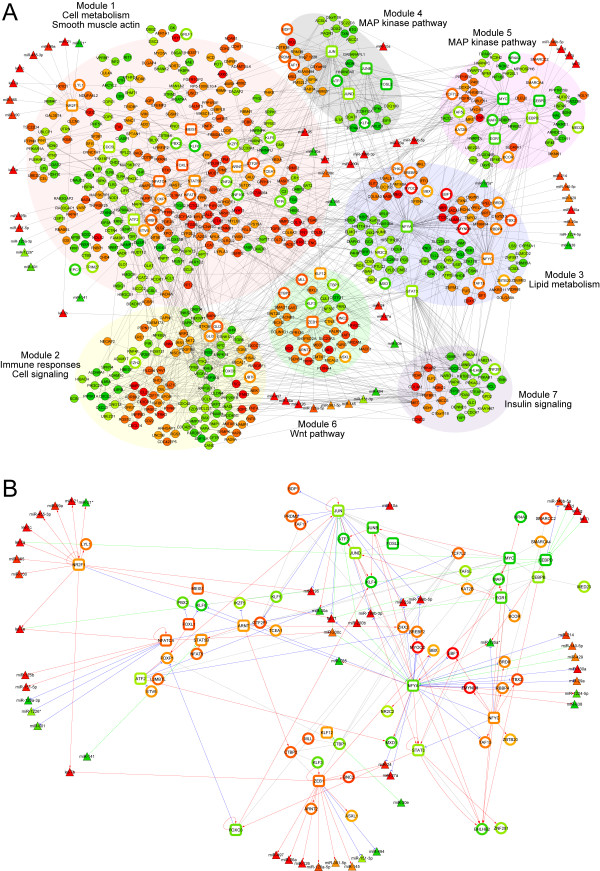
**A hypothetical network based on differentially expressed transcription factors and DEmiRNAs**. Molecular interactions obtained from protein-protein interactions, KEGG pathway interactions, putative transcriptional regulatory interactions derived from 1423 DEGs, and predicted DEmiRNA and DETF interactions were combined. (A). The network containing 689 nodes composed of 22 DETFs (open squares), 618 DEGs (filled circles) and 49 DEmiRNAs (filled triangles) and 1391 non-redundant interactions was generated. The network can be grouped into 7 modules (as indicated) based on the connectivity of nodes - the more interacted nodes are grouped together. (B) This figure displays only the network consisting of the DETF (with known binding sites) and their cognate DEmiRNAs. The transcription factors, which do not have well characterized binding site information were not used in prediction of interactions, and are shown as open circles.

Combining predicted DETF-DEmiRNA and DETF-DEG transcriptional interactions based on conserved transcription binding site information and expression profiles with protein-protein interactions and curated interactions in the KEGG database, we built a molecular network centered on twenty-two well-characterized DETFs (see Additional file 1 for details). There are a large number of DEmiRNA-DEG (non-DETF) interaction pairs (681 pairs having FDR<0.05 and 1470 pairs having FDR<0.1, see Additional file 1), and the effects of the DEmiRNA-DETF interactions are expected to be key to regulation of many other genes. Thus, we decided first to focus on the effects of the DEmiRNA-DETF interactions. The entire network (excluding DEmiRNA-DEG interactions) is still large and contains 689 nodes (562 DEGs, 49 DEmiRNAs and 78 DETFs) with 1391 interactions (Figure 4). Out of the 22 DETFs, 17 have predicted interactions with DEmiRNAs. The majority of the DETF-DEmiRNA interactions are centered on 6 TFs, which represent the core of the network. They are nuclear receptor subfamily 2, group F, member 1 (*NR2F1*), *JUN*, nuclear factor of activated T-cells, cytoplasmic, calcineurin-dependent 4 (*NFATC4*), zinc finger E-box binding homeobox 1 (*ZEB1*), nuclear transcription factor Y, alpha (*NFYA*) and CCAAT/enhancer binding protein (C/EBP), delta (*CEBPD*). Based on the network topology, this global DEG/DETF/DEmiRNA interaction network could be grouped into seven large modules (labeled as 1 to 7 on Figure 4a). We also attempted to sub-group the networks based on KEGG pathways but the attempt was not very successful, since there are too many shared genes among different "pathways" (Additional file 7). Though the pathway-based analysis has some disadvantages relative to a purely topological approach, it revealed that there are 84 genes in the network related to signal transduction and 69 genes involved in the immune system. This suggests the strong involvement of immune response and cell-cell communication related pathways in processes associated with ILDs. The GO term and KEGG pathway enrichment analyses revealed that genes in module 1 are enriched with metabolic-related terms and pathways. In addition, module 1 also contained all the smooth muscle-actin-related genes from the vascular smooth muscle contraction pathway. Module 2 contained genes involved in the immune response, cellular signaling process and cell-cell communication. Module 3 contained genes involved in lipid metabolism, and module 7 contained genes associated with adipocytokine and insulin signaling processes. The Wnt pathway is associated with module 6, while the MAPK pathway is associated with module 4 and 5 (Table 3 and Additional file 1).

**Table 3:** Pathways associated with different network modules.

			Module						
			
		KEGG Pathway	1	2	3	4	5	6	7
Metabolism	Amino Acid Metabolism	Cysteine and methionine metabolism	7E-02						
		Glycine, serine and threonine metabolism	5E-02						
		Valine, leucine and isoleucine degradation	**8E-03**						
	
	Biosynthesis of Secondary Metabolites	Terpenoid backbone biosynthesis	**8E-03**						
	
	Carbohydrate Metabolism	Butanoate metabolism	7E-02						
		Inositol phosphate metabolism		1E-02					
		Propanoate metabolism	7E-02						
		Pyruvate metabolism	3E-02						
	
	Energy Metabolism	Reductive carboxylate cycle (CO2 fixation)	3E-02						
	
	Glycan Biosynthesis and Metabolism	Glycosaminoglycan degradation	**2E-03**						
		Glycosphingolipid biosynthesis - ganglio series	7E-02						
		Heparan sulfate biosynthesis	4E-02						
	
	Lipid Metabolism	Arachidonic acid metabolism	2E-02						
		Ether lipid metabolism	8E-02						
		Fatty acid elongation in mitochondria	2E-02						
		Fatty acid metabolism	3E-02						
		Glycerolipid metabolism	**2E-03**						
		Glycerophospholipid metabolism	1E-02						
		Sphingolipid metabolism			2E-02				
		Steroid biosynthesis			***8E-05***				
		Synthesis and degradation of ketone bodies	3E-02						
	
	Metabolism of Cofactors and Vitamins	Nicotinate and nicotinamide metabolism	**5E-04**						
		One carbon pool by folate	7E-02						
	
	Metabolism of Other Amino Acids	Selenoamino acid metabolism	4E-02						
	
	Xenobiotics Biodegradation and Metabolism	Benzoate degradation via CoA ligation	6E-02						
		Caprolactam degradation	**8E-03**						

Genetic Information Processing	Folding, Sorting and Degradation	RNA degradation					2E-02		

Environmental Information Processing	Signal Transduction	ErbB signaling pathway		2E-02					
		Hedgehog signaling pathway		***4E-05***					
		Jak-STAT signaling pathway							1E-02
		MAPK signaling pathway		8E-02		**5E-03**	**2E-03**		
		mTOR signaling pathway		1E-02					
		Notch signaling pathway						**4E-03**	
		Phosphatidylinositol signaling system		4E-02					
		TGF-beta signaling pathway					4E-02	1E-02	
		VEGF signaling pathway	6E-02	**9E-03**					
		Wnt signaling pathway		1E-02				**1E-04**	
	
	Signaling Molecules and Interaction	Cell adhesion molecules (CAMs)						3E-02	
		Cytokine-cytokine receptor interaction		**4E-03**					6E-02
		ECM-receptor interaction	**1E-02**						

Cellular Processes	Cell Communication	Adherens junction							3E-02
		Focal adhesion	1E-02	**5E-04**					3E-02
		Tight junction		**4E-04**					
	
	Cell Growth and Death	Apoptosis		2E-02					
		Cell cycle					8E-02		
		p53 signaling pathway		3E-02					
	
	Cell Motility	Regulation of actin cytoskeleton		**9E-04**					
	
	Circulatory System	Vascular smooth muscle contraction	***4E-06***						
	
	Development	Axon guidance		7E-02					7E-02
	
	Endocrine System	Adipocytokine signaling pathway		3E-02					***2E-06***
		GnRH signaling pathway		9E-02					
		Insulin signaling pathway		***2E-05***			1E-02		***7E-05***
	
	Immune System	Antigen processing and presentation			8E-02				
		B cell receptor signaling pathway		**8E-03**					
		Chemokine signaling pathway		***2E-09***					
		Complement and coagulation cascades	1E-02						
		Fc epsilon RI signaling pathway		4E-02					
		Fc gamma R-mediated phagocytosis		**1E-03**					
		Leukocyte transendothelial migration		1E-02					
		T cell receptor signaling pathway		**2E-03**					
		Toll-like receptor signaling pathway	2E-02	9E-02					
	
	Nervous System	Neurotrophin signaling pathway		***7E-06***					
	
	Transport and Catabolism	Endocytosis		**3E-04**					

Human Diseases	Cancers	Acute myeloid leukemia					2E-02		
		Basal cell carcinoma		***3E-05***					
		Chronic myeloid leukemia		**8E-03**				**9E-03**	
		Colorectal cancer		1E-02			4E-02		3E-02
		Endometrial cancer		6E-02			1E-02		
		Glioma		1E-01					
		Pancreatic cancer		3E-02					
		Pathways in cancer		***3E-07***				**3E-03**	
		Prostate cancer					4E-02		
		Renal cell carcinoma		3E-02					
		Small cell lung cancer		1E-02					
		Thyroid cancer					**5E-03**		
	
	Circulatory Diseases	Arrhythmogenic right ventricular cardiomyopathy (ARVC)	6E-02						
		Hypertrophic cardiomyopathy (HCM)							**2E-04**
	
	Infectious Diseases	Epithelial cell signaling in Helicobacter pylori infection		***2E-05***					
	
	Neurodegenerative Diseases	Amyotrophic lateral sclerosis (ALS)		6E-02					

With genes in each module of the network, pathway enrichment analysis was performed using one-sided Fisher exact test. The significance level of p-value is represented by regular (0.01~0.1), bold (0.0001~0.01) and bold-italic (<0.0001) fonts.

### Transcription factors and miRNA may co-regulate biological networks

The most direct effects of miRNAs are through the specific interaction with their mRNA targets, affecting the stability of these mRNAs or attenuating protein translation. The more complex and broad indirect effects of miRNAs occur when they engage regulatory networks by modulating the levels of specific TFs. These effects are more numerous, pervasive, and difficult to untangle. For example, the mRNA for *NFYA *is a predicted target for miR-429, and the level of this mRNA may therefore be affected by the level of miR-429 (Figure 4b). Since *NFYA *regulates many other genes and miRNAs, a large biological network may be affected by this specific miRNA-TF connection. In addition, miRNAs may work in conjunction with TFs to modulate the activity of various molecular processes. Examples of this type of more complex regulatory circuit, feed-forward loops (FFL), have been described and analyzed in *E. coli *and yeast [38,39]. A FFL has at least two branches, with either activation or suppression toward its targets. It is called a coherent FFL when all the regulatory signals are synergistic; otherwise, it is termed an incoherent FFL [40]. They can have specific regulatory characteristics, for example a coherent FFL effectively filters out rapid fluctuations in the inputs.

**Figure 5:**
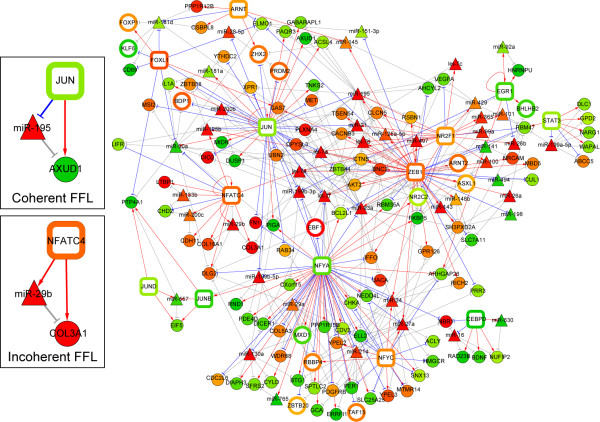
**Examples of potential feed forward regulatory loops**. A potential feed forward loop (FFL) was generated if a DEmiRNA and its predicted regulatory transcriptional factor targeted the same DEG (see Additional file 1). Red, blue and gray lines represent the predicted transcriptional activation (i.e. positive correlation), predicted transcriptional repression (i.e. negative correlation) and relationships between DEmiRNAs and their predicted targets (without considering expression correlations), respectively. The left inserts illustrate coherent and incoherent FFLs. JUN, miR-195 and AXUD1 form a coherent FFL in which transcriptional (from JUN to AXUD1) and miRNA-mediated regulation (from JUN to AXUD1 via miR-195) are synergistic. In contrast, NFATC4, miR-29b and COL3A1 form an incoherent FFL in which two opposite regulatory interactions occur.

To identify FFLs composed of DEmiRNAs, DETFs, and DEGs, we compiled all putative interactions among them and applied statistical filters (FDR<0.1 for all DETF-DEmiRNA and DETF-DEG interactions, see Additional file 1). Among the entire 170 nodes (13 DETFs and 46 DEmiRNAs with 111 DEGs) that constituted the FFL network, *JUN*, *NFYA*, *ZEB1*, and miR-23a emerged as the top 4 most "connected" nodes (Figure 5). For example, *JUN*, miR-195 and *AXUD1 *consist of a coherent FFL since regulatory effects of *JUN *and miR-195 would be expected to act together to decrease the level of *AXUD1 *protein expression. On the other hand, *NFATC4*, miR-29b and *COL3A1 *represent an incoherent FFL since *NFATC4 *and miR-29b regulate *COL3A1 *in the opposite directions. Because of the potential for significant regulatory impact of the EMT process, we explored in more detail one of the predicted FFLs that includes *ZEB1 *and the mir-23 miRNA cluster.

### Identification of additional regulatory factors that influence EMT

*ZEB1 *encodes a key transcription factor that acts downstream of TGF-β which has been implicated in EMT [41]. We observed elevated levels of Zeb-1 mRNA expression in our ILD samples (Figure 4A, B; Figure 5) which led us to examine its role downstream of TGF-β in more detail. Zeb-1 has 19 direct interacting DEmiRNAs, based on predicted interactions. As with Zeb-1, the levels of a number of these miRNAs, especially miR-23a, miR-24, miR-26a, and miR-27a were elevated in the ILDs. These miRNAs are encoded at a single genomic locus on chromosome 19p13, the miR-23a cluster. Further, we noted that there are two E-box motifs, or presumptive binding sites for Zeb-1, in the distal promoter of the miR-23 locus (Additional file 8). Thus, we explored the possibility that Zeb-1 regulates or co-regulates the transcription of miRNAs in the miR-23a cluster.

**Figure 6:**
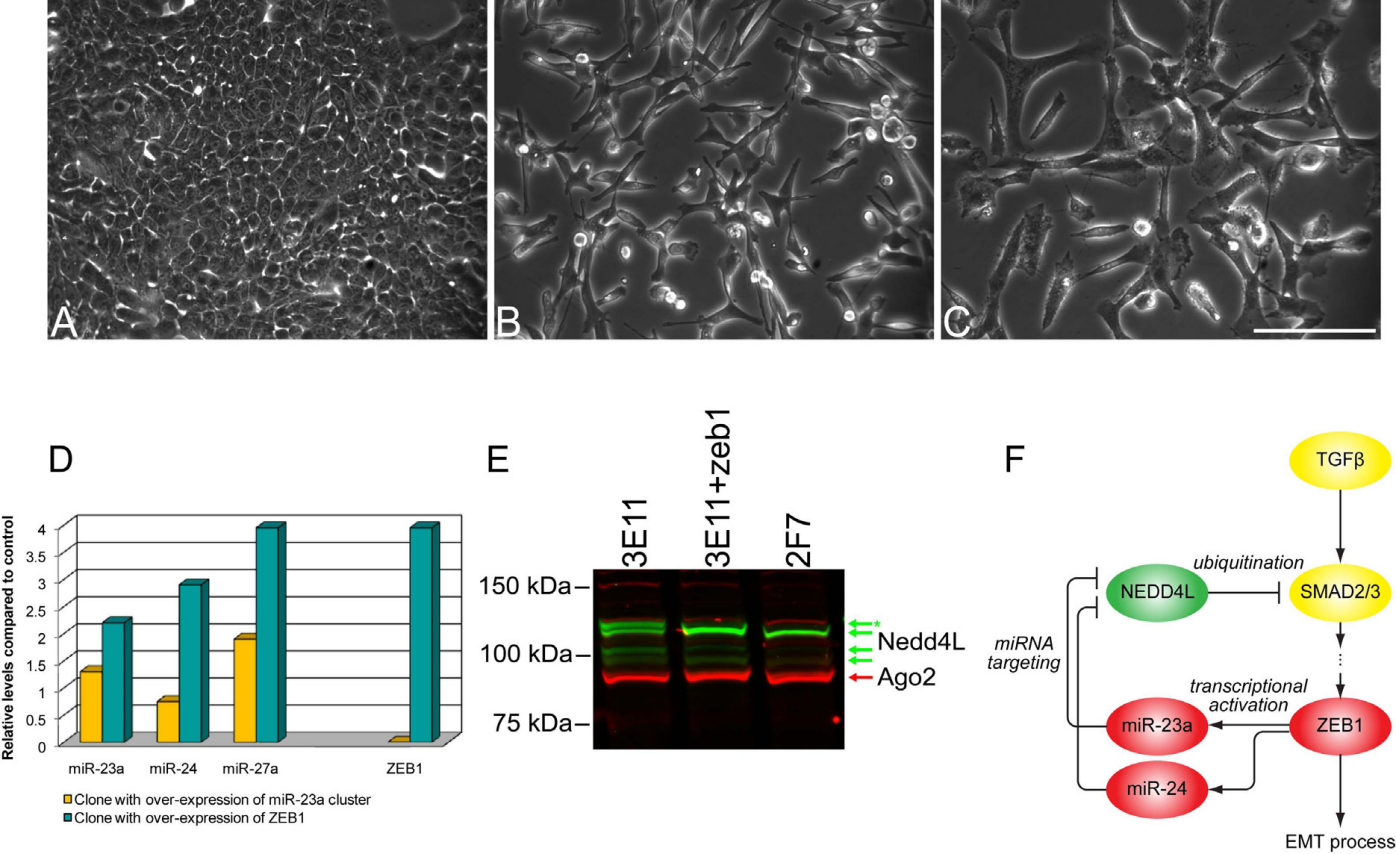
**The Zeb-1 mediated EMT is partially regulated by miRNAs in the miR-23a cluster**. (A-C) Phase contrast images showing the morphology of an epithelial MDCK clone (A; 3E11), the same epithelial clone stably over-expressing Zeb-1 (B; 3E11+Zeb1), or a mesenchymal MDCK clone (C; 2F7). (D) Over-expression of Zeb-1 causes the levels of miR-23a, miR-24 and miR-27a to rise dramatically, while over-expression of the miR-23a cluster of genes had no effect on the level of Zeb-1 mRNA, relative to mock-transfected cells. (E) Nedd4L Western: MDCK clone 3E11 has an epithelial morphology and expresses 4 bands that are recognized by an anti-Nedd4l antibody (green). One of these bands (marked with asterisk) was dramatically reduced in MDCK cells with mesenchymal morphology (clone 2F7) or in an epithelial MDCK cell line stably over-expressing Zeb-1 (3E11+Zeb1). The significance of each of the four bands recognized by the anti-Nedd4L antibody remains unclear. However, a similar pattern of bands was observed using a second anti-Nedd4L antibody (data not shown). Anti-Argonaute 2 (Ago2; red) antibody was used as loading control. (F) A hypothetical model for the role of the Zeb-1, the miR-23a cluster, and Nedd4L in TGF-β mediated EMT. The genes and miRNAs involved in the process are listed and the colors indicate the relative expression changes in ILD samples compared to control, red indicates higher level in ILDs, yellow indicates no significant changes, while green represents lower levels in ILD samples compared to control.

To test whether Zeb-1 can influence the induction of the miR-23a cluster and to investigate what the consequences might be, we used an *in vitro *EMT model in the well-characterized Madin-Darby canine kidney (MDCK) epithelial cell line. These cells normally express high levels of E-cadherin, other epithelial cell markers, and a low level of Zeb-1 and they exhibit the morphology of an epithelial cell (Figure 6A) An increase in Zeb1 expression results in an EMT [41] leading to a mesenchymal morphology (Figure 6B). Separate MDCK cell lines (derived by us from single-cell clones) were constructed in which either Zeb-1 or the miR-23a cluster was over-expressed. Over-expression of Zeb-1 caused a dramatic increase in the levels of all the miRNAs that make up the miR-23a cluster, while over-expression of these miRNAs had no such effect on Zeb-1 mRNA as shown in Figure 6D. This is consistent with the miR-23a cluster being positively regulated at the level of transcription by Zeb-1. We next explored which possible targets of the miR-23a cluster might be part of this signaling pathway.

Through computational target prediction, 'neural precursor expressed, developmentally down-regulated protein 4-like' (*NEDD4L*), which showed significantly reduced expression in ILD (-2.46 fold) was identified as one of the targets for the members of the miR-23a cluster. Indeed, high levels of Nedd4L protein are readily detected in MDCK cells that have an epithelial morphology. There are several forms of Nedd4L protein present, as reported in the literature. The predominant form of Nedd4L detected in our Western blot is distinctly different between MDCK cell clones with epithelial or mesenchymal morphology, or in epithelial MDCK cells overexpressing Zeb-1 (Figure 6E). Nedd4L, a ubiquitin ligase, has been shown to down-regulate TGF-β pathway activity, possibly by triggering Smad2/3 and TGF-β type I receptor (Tgfbr1) ubiquitination and proteasome-dependent degradation [42,43]. We are therefore currently testing the role of Nedd4L in ILD processes. Lower levels of Nedd4L in ILDs may result in higher TGF-β signaling activity in these tissues. Together, these findings suggest that Zeb1-mediated EMT, through mir-23a cluster regulation of Nedd4L protein levels, may stabilize or enhance the activity of TGF-β signaling and thus contribute to disease persistence (Figure 6F).

### Integrated biological networks can be used to classify pathology

**Figure 7:**
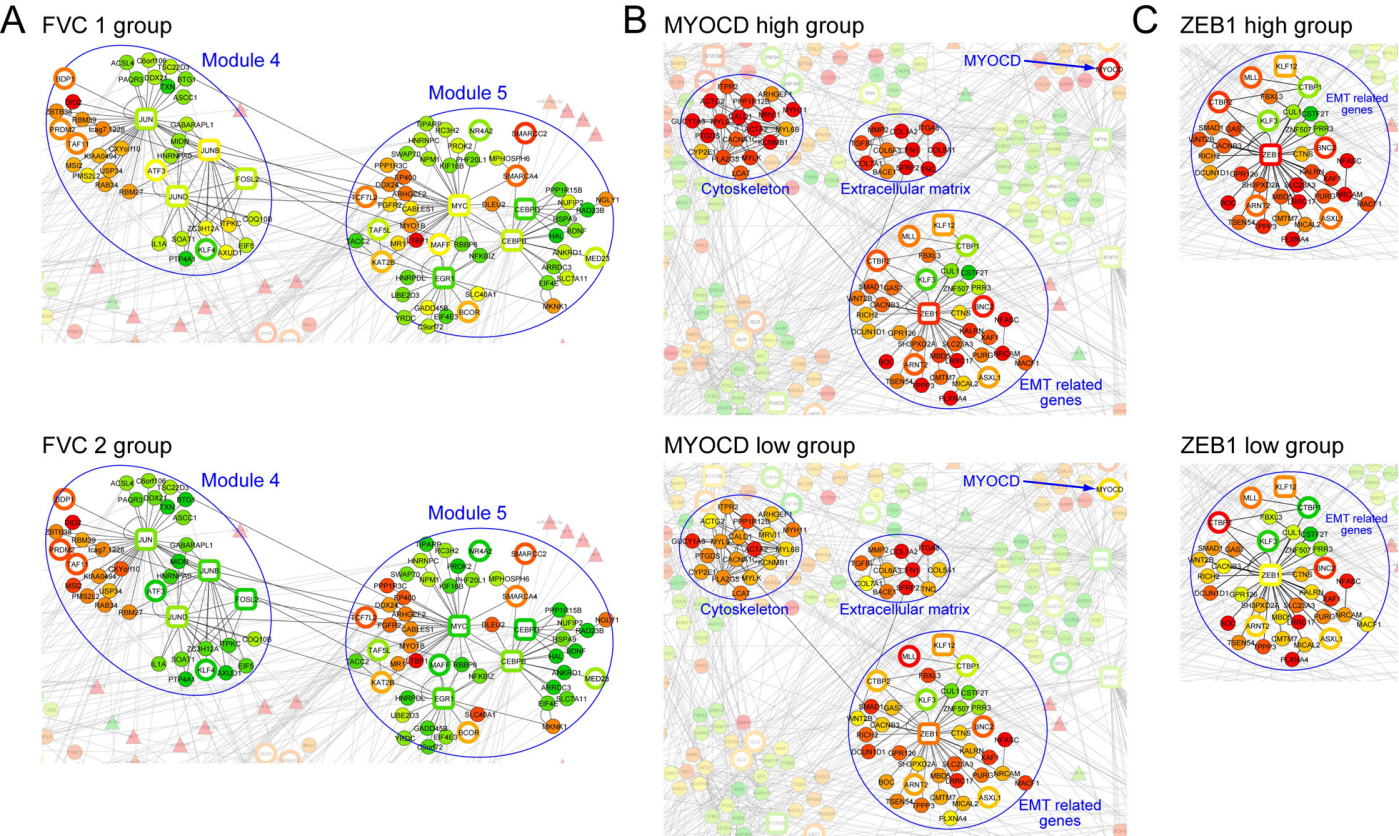
**Examples of sub-networks associated with different patient classifications**. Examples of sub-networks that show significant changes in some key genes associated with FVC groups 1 and 2 (A), MYOCD (B), or ZEB 1 (C) expression levels. The corresponding FVC group and MYOCD or ZEB 1 genes are labeled in the sub-networks. The levels of expression are indicated as in the previous figures.

A key reason to build integrated molecular network models is to understand the pathological changes at a molecular level between normal and ILD states. However, only some of the modules defined in Figure 4 varied among different FVC groups or subtypes of ILDs. Modules 4 and 5 showed changes between FVC group 1 and group 2; specifically the transcription factors in these modules showed 2 to 8 fold increases from the FVC group 2 to the FVC group 1 (Figure 7a). Genes associated with signaling pathways are enriched in these two modules (Table 3). Except between FVC group 1 and FVC group 2 in modules 4 and 5, we could not identify significant changes at the network level either between the FVC group 2 and FVC group 3, or between patient groups with different diagnoses.

However, when we grouped the patients based on the expression levels of key molecules having roles in the progression of disease, such as *MYOCD *or *ZEB1 *(see Additional file 1), several significant correlations were identified. In patient samples with high *MYOCD *expression levels, we observed significantly higher levels of DEGs related to smooth muscle cytoskeleton, the extracellular matrix and the EMT process (Figure 7b). This observation correlates well with the hypothesis that a higher level of *MYOCD *expression is linked to a higher number of fibrocytes in the tissue. In the case of Zeb1, there were significant changes in the levels of DEGs that putatively interact with Zeb1 mRNA or protein (module 6 in Figure 4A). Interestingly, the levels of several Kruppel-like transcription factors (KLF) including *KLF3*, *KLF4*, *KLF6 *and *KLF9 *in ILD patients paralleled changes with the *ZEB1 *(higher levels of ZEB1 with lesser decreases in the KLFs). The fact that changes in some modules are apparently associated with different levels of key genes involved in the ILDs suggests that it may be valuable to use molecular profiling and network modeling in order to group and stratify patients which could aid in better understanding and treating ILDs.

## Discussion

While the earliest inciting events that result in ILDs remain unclear, IPF and some closely related interstitial lung diseases share a clinical course in which a zone of fibrosis expands at the expense of alveolar structures [1,4]. The relentless expansion of fibrosis and the failure of re-epithelialization suggest that the initiation, maintenance, and resolution of the tissue repair process in ILD lungs are probably changed in some fundamental ways. To understand these processes in more detail, we investigated expression of mRNA and miRNA in human lung biopsy specimens from patients with ILDs and compared the findings with control lung samples to identify the networks and regulatory modules involved in disease.

Global transcriptome analysis, including miRNAs, is a useful tool to investigate how molecular networks change between biological states. However, caution is called for when interpreting these data. RNA profiling methods measure the concentrations of RNA (for both mRNA and miRNA) at a specific state; dynamically modulated processes and transcripts can be missed. In addition, this approach cannot detect regulatory changes associated with translation of messages. Nor do the levels of miRNAs always correspond to their regulatory activities, which can be modulated by other proteins or RNAs. Significant variations in measurements can also come from sample heterogeneity and sample preparation, such as the difference in biopsy locations, the heterogeneity of the disease within the biopsy sample, the diverse spectrum of cell types in the sample, and genetic and environmental effects on individuals. Finally, the profiling methods used in the study can only observe the average effect of multiple cell types and numbers of cells in different states in the sample which can add uncertainly to the data. The generally modest correlation among different miRNA measurement platforms [44] also needs to be taken into consideration when comparing data from other studies. Nonetheless, with all these qualifications, global mRNA/miRNA profiling is currently the most powerful available approach to identify disease associated molecular networks.

By analyzing mRNA and miRNA spectra, we identified 1423 differentially expressed genes and 125 differentially expressed miRNAs between ILD and control lung samples. Comparing the data from three male and three female control lung samples, females generally exhibit lower (albeit modestly) levels of expression of both mRNA and miRNA than males. There are exceptions, however, for example, the average levels of miR-101 and miR-27b are 8 times higher (per cell) in males than in females, and Atlastin3 (*ATL3*) and Sec23 homolog A (*SEC23A*) mRNA are three times higher in males when compared to females. Unfortunately, we do not have sufficient numbers of samples to determine whether these gender differences play any role in the progression of ILDs. Interestingly, gender-associated pathological development has been observed in other lung conditions [45,46]. It is certainly worth further investigation in the future with larger, carefully age- and gender-matched sample sets.

Regardless of gender, our data also indicates a general inverse trend in global gene expression patterns as the severity of the disease increases and as lung function decreases (Figure 1). For example, mRNAs for the transcription factors, *MYC *and *JUNB *show significant differences in samples from patients in FVC group 3 (lung function greater than 80% of normal) and group 2 (lung function between 50 to 80% of normal), but almost no change in samples from FVC group 1 patients (lung function less than 50% of normal) when compared to controls. This contradicts the general expectation of larger changes in more advanced disease. However, in ILDs there is significant tissue remodeling and functional adaption. Individuals with the most severe disease, FVC group 1, may adapt to the deficiency of lung capacity, and the repopulation of the lung parenchyma with fibroblasts may also mask the signals from stressed pulmonary epithelial cells, which are proportionally a smaller and smaller fraction of the tissue as disease progresses.

### Pairs of genes and miRNAs can be used to distinguish different disease types

Biomarkers that help diagnose the ILDs or that can distinguish idiopathic pulmonary fibrosis from other forms of ILDs, or reflect the severity of a disease would be useful tools to combine with other clinical information such as patient histories, pulmonary function tests, and radiographic findings to achieve better diagnosis of the diseases. Using "top scoring pair analysis", we identified several pairs of mRNAs and microRNAs that had the power to discriminate ILDs from controls (see Additional files 4 and 5). The most robust pairs, *COL3A1 *and *ARHGEF7*, and miR-99b and miR-21*, can perfectly discriminate the ILD samples from controls (Additional file 5) in our sample set. Using the same approach, we also identified mRNA pairs that distinguish IPF from NSIP (Additional file 5). Even though these mRNA or miRNA pairs can successfully distinguish ILDs from normal or separate UIP/IPF from NSIP, these candidate markers were derived from lung tissue biopsies, which are not routinely used in the clinic; as a result, their immediate value may be limited.

### Several pathways are significantly perturbed in disease

As in previous studies, our transcriptomic study also revealed a number of strongly perturbed pathways in the ILDs. These include TGF-β, Wnt signaling, focal adhesion, extracellular matrix-receptor interactions, insulin signaling, MAPK signaling, B-cell signaling, adipocytokine signaling, and vascular smooth muscle contraction pathways [4,7,9]. The involvement of these pathways clearly suggests the involvement of the immune response and a disruption of the normal cellular microenvironment in the ILDs. We also observed that a number of metabolic processes such as glycine, serine and threonine metabolism and steroid biosynthesis were suppressed in the ILD samples, which implies a decrease in general lung metabolic activities in ILD patients. Consistent with this finding from DEGs, we also observed changes in several immune response related miRNAs including miR-100, mir-21, miR-140, miR-146, miR-155, and miR-223 [47,48].

An interesting finding of the pathway enrichment analyses is the similarity of the differentially expressed mRNA we observed with the predicted target mRNAs of the differentially expressed miRNAs (Table 2). This suggests that the miRNAs we observed may have key roles in regulating the basic pathological processes of the ILDs as illustrated by pathways for Wnt signaling, ECM-receptor interaction, focal adhesion, vascular smooth muscle contraction, adipocytokine signaling and insulin signaling.

### Focusing on transcription factors and miRNAs provides an incisive view of regulatory networks associated with ILDs

Even though pathway enrichment analysis with DEGs and DEmiRNA targets allowed us to identify molecular pathways associated with ILDs, this approach has two limitations: 1) it limits us to previously curated pathway information and 2) it lacks sufficient information to build an integrated view associated with ILDs from the dataset. We thus focused on the transcription factors and miRNAs, to provide a global view of the regulatory machinery involved in the ILDs. We computed a network with 640 DEGs, and 49 miRNAs with a total of 1391 interactions (excluding DEmiRNA-DEG interactions). Compared to a simple pathway-based analysis, in which only 395 of the 1423 identified DEGs could be assigned to any pathway, this transcription factor and miRNA-mediated network analysis allowed us to capture and integrate much more information. It is important to realize however, that until we can measure the cognate protein levels for all the relevant genes in these networks, the translation regulation component of the network will remain hidden. This component undoubtedly plays an important role in the disease-related network regulation, and so it must be a priority for future work to assess the protein levels for all the relevant genes if we are to understand the perturbation of these gene networks more completely.

Grouping the most highly interacting nodes together [49], the network can be grouped into 7 modules. It is encouraging that the biology is reflected in this grouping, since the modules align with functional associations based on pathway and GO term enrichment analyses (Table 3 and Additional file 1). For example, module 1 contains genes from a number of metabolic pathways, and module 2 contains genes involved in immune responses and signal transduction processes (Table 3). This approach also allows us to integrate various molecular processes into a single module and reveal key molecules linking these processes. For example, *CDC42*, phospholipase C, beta 4 (*PLCB4*), mitogen-activated protein kinase 14 (*MAPK14*), and Src homology 2 domain containing transforming protein 3 (*SHC3*) are all located in module 2 and these gene products are clearly involved in the receiving and transmission of various extracellular signals to the nucleus.

Because of their documented functions, genes like *MYOCD *or *ZEB1 *may be used as indicators of EMT or the presence of mesenchymal cells such as myofibroblasts in the ILD samples. Indeed, when we grouped patients based on the expression levels of these genes, several groups of genes related to *MYOCD *or *ZEB1 *were revealed. In addition to the obvious sub-networks representing changes in the cytoskeleton, the extracellular matrix and the process of EMT (Figure 7), several key transcription factors such as the zinc-finger KLF family members, *JUN*, *MYC*, *EGR1*, and *MEIS1 *also showed significant changes associated with the *MYOCD *and *ZEB1 *mRNA expression levels. The involvement of these transcription factors and their effects on the ILDs warrants further study with more patient samples and different clinical pathologies.

Another reason to build an integrated molecular network associated with ILD is to gain a better understanding of the overall pathology. In most cases we could not observe a clear association between the changes of network modules with different clinical conditions such as FVC groups or diagnosed subtypes of ILDs. This most likely reflects diversity in the ILD pathologies, the differing degrees of progression and especially the difficulty of making precise and consistent clinical observations embodied in the recorded diagnoses. This further illustrates the need for new approaches like those used here, such as using the levels of *ZEB1 *and *MYOCD *mRNAs to provide a more consistent and accurate clinical stratification in this complex of diseases.

### Anti-apoptosis signal may promote survival of myofibroblats in ILD lungs

The lung tissues from ILDs in general, and from IPF in particular, are enriched with myofibroblasts. Myofibroblasts utilize their actin-linked cytoskeletal machinery to migrate, proliferate, form foci and perhaps contract under stimulation. A series of up-regulated genes, from calcium and potassium regulated channels located at the cell membrane (calcium channel, voltage-dependent, L type, alpha 1C subunit (*CACNA1C*) and potassium large conductance calcium-activated channel, subfamily M, beta member 1 a (*KCNMB1*)), to the cytosolic genes that regulate the levels of secondary messengers (cyclicGMP, cyclicAMP, diacylglycerol, Ca^++^, inositoltriphosphate) to myosin light chain kinases, (*MYLK *and *MYLK3*) actins, and myosins (*MYL9*, *MYH11*, *ACTA2*, *ACTG2*), outline key molecular pathways and constitute an unmistakable signature of the myofibroblast. These genes are clustered on the lower right corner of module 1 (Figure 4a). The apparent monolithic expression changes of genes associated with α-smooth muscle networks are in agreement with the abundance of myofibroblasts in the biopsy tissue we studied. The strong down-regulation of several apoptosis related genes such as receptor-interacting serine-threonine kinase 1(*RIP1*), bcl2-associated X protein (*BAX*), and phosphoinositide-3-kinase (*PI3K*) may also contribute to the resistance to apoptosis of the myofibroblasts.

### miRNAs from the miR-23 cluster may mediate Zeb1 induced epithelial to mesenchymal transition through modulating TGF-β activity

The origin of the fibroblasts in the ILD lung could come from three major sources--the proliferation of resident pulmonary fibroblasts, the recruitment of circulating fibrocytes into the lung and EMT of pulmonary epithelial cells. Zeb-1 has been shown to be a positive regulator of mesenchymal cell related genes; it can participate both in epithelial to mesenchymal as well as a mesenchymal to epithelial transitions [50]. Prolonged exposure to TGF-β has been seen to induce expression of the transcription factors Zeb-1 and Zeb-2, leading to EMT. In addition to other targets, Zeb-1 and Zeb-2 are known to negatively regulate expression of the miR-200 family. These miRNAs have been proposed to maintain an epithelial phenotype in some cells and their decline in response to Zeb-1 commits the cells to transition to a mesenchymal phenotype [51-54]. In our ILD samples, we observed a significant increase of Zeb-1 mRNA levels which is consistent with a role in EMT in the ILD. Surprisingly, we also observed an increase in the miR-200 family in our ILD samples. These observations raise the possibility that both processes could be active in our lung tissue biopsies, but in different cells: negative regulation of EMT mediated by miR-200 family in epithelial cells and positive regulation of EMT, mediated by TGF-β, Zeb-1, and the miR-23 cluster, in transitional cells and mature fibroblasts.

Our network analysis also suggested a preliminary model for how a signal from TGF-β could result in transcriptional and post-translational regulation that would lead to EMT and maintenance of the mesenchymal myfibroblast compartment in ILD. Zeb-1 mRNA expression was up-regulated in ILD as was the expression of miR-23a, miR-27a and miR-24. These miRNAs are encoded at one locus in the human genome at 19p13 and constitute the miR-23a cluster. The similar expression profile of these miRNAs in our ILD data suggested that Zeb-1 may positively regulate this cluster of miRNAs. Putative binding sites for Zeb-1 proximal to the cluster support this hypothesis (see Additional file 8). We investigated this relationship further by over-expressing Zeb-1 in MDCK cells *in vitro*, which confirmed this suggestion: Zeb-1 clearly caused the levels of the miR-23a cluster to increase (Figure 6D). In addition, we identified the ubiquitin ligase Nedd4L as likely being subject to post-transcriptional negative regulation by the miR-23a cluster, which is driven in turn by over-expression by Zeb-1. The changes in Nedd4L expression are shown in Figure 6E. We believe the presence of multiple Nedd4L isoforms in the Western blot is due to the combined effect of alternative start codons and alternative splicing events (the detailed analysis of this phenomenon will be reported elsewhere). It is also possible that these results, based on the use of a kidney cell line, may not be applicable in all details to the lung. Since Nedd4L accelerates the degradation of Smad2/3 and Tgfr1, thereby attenuating TGF-β signaling [42,43], the Zeb-1 feed forward loop (FFL) we identified here is likely to be a component of the larger pathway by which TGF-β signaling contributes to EMT, and thus to expansion and persistence of the myofibroblast compartment in ILD. In susceptible pulmonary epithelial cells, TGF-β signals *via *Smad2/3 and 4 to upregulate Zeb-1, which in turn promotes the transcription of the miR-23a cluster of miRNAs. These miRNAs suppress Nedd4L activity at a post-transcriptional level, resulting in uninterrupted signaling activity from Smad2/3 (Figure 6F). We believe it should be possible to validate this aspect of TGF-β signaling in myofibroblasts derived from ILD tissue. It illustrates the utility of network predictions based on comprehensive transcriptome data, and suggests a promising line of further investigation.

Recently, Pandit et al. [55] reported that the level of let-7d, regulated by TGF-β signaling, may play a role in EMT and contribute to IPF pathogenesis. This conclusion was partly based on the observed increase of mesenchymal markers including *CDH2*, *VIM *and *ACTA2 *relative to let-7d levels. We also have observed perturbation of the TGF-β pathway (Table 3, Figure 4a. module 5 and 6), over-expression of *ZEB1 *(Figure 4A, module 6), and increased levels of mesenchyme-associated genes such as *ACTA2*, *CDH2 *and *VIM *in ILD patients (Figure 4A, module 1). Although Pandit et al. and our study agree on the observation of mesenchymal markers and the lack of a significant decrease in the miR-200 family, we did not observe a decrease in let-7d in the ILD patient samples in this study. This difference could be due to the fact that our patient population has a more diverse set of diagnoses, even though none of the IPF patients in our study were observed to exhibit this decrease, or to different miRNA quantitation methods. These overall findings support the idea of multiple parallel processes involved in perturbing key biological pathways or networks in the pathogenesis of ILD.

## Conclusions

Systems biology approaches yield a much more complete picture of how signaling networks and groups of co-expressed genes are involved in ILDs, which may help to explain key aspects of disease pathogenesis. The transcription factor and miRNA-based network analysis allows us to gain a global view of the molecular control networks associated with ILDs. It also provides new insights into the diseases and generates new testable hypotheses, such as how Zeb-1 activity is partially mediated through the miRNAs in the miR-23a cluster (Figure 6F). Our network and pathway analyses helped clarify two processes that are fundamental to ILD pathogenesis: the strong anti-apoptosis signal and the origin of myofibroblasts via EMT. Taken together, these processes help explain why the progressive pro-fibroblast environment fails to resolve. We validated the possible involvement of the miR-23a cluster in modulating TGF-β activity to facilitate the Zeb1-mediated EMT process. This finding may provide new therapeutic targets for intervention, such as anti-miR therapeutics delivered to the lung to decrease Zeb-1 activity. Another interesting possibility is specifically to induce apoptosis of fibroblasts.

We have demonstrated here the power of focusing on the key regulatory components, the transcription factors and miRNAs, in order to gain insight into the networks perturbed in complex diseases such as ILDs. The inferred network: 1. provides a global view of the effects of DETFs and DEmiRNAs, 2. is not limited to individual molecular processes or pathways, and 3. uses information beyond curated databases to link molecules and networks. Despite the apparent conceptual simplicity of this approach, it is difficult to construct such networks, and most of the miRNA and TF mediated interactions are not yet well-characterized. In addition, there is no definitive method by which to modularize the network so that functional implications can be easily viewed and extracted. Our approach therefore needs further development in data integration and visualization; nevertheless, it represents a significant step in the global view of perturbed network in the complex of interstitial lung diseases. Lacking longitudinal time-course samples or samples accurately matched to disease state progression prohibits following the dynamic changes in ILD. Temporal dynamics is one of the most powerful elements needed to fully unravel the biology of a system. Integrating data from properly chosen model systems should allow us to gain a better understanding of disease initiation and progression. We would expect that some of the same networks are perturbed in these models, and allow us to build better network-based molecular models to identify, test and predict new therapeutic interventions.

## List of abbreviations

DEG: differentially expressed gene; DEmiRNA: differentially expressed microRNA; DETF: differentially expressed transcription factor; EMT: epithelial to mesenchymal transition

## Competing interests

The authors declare that they have no competing interests.

## Authors' contributions

DG, CM, RG, KW, MP planned the experiments; JP, SZ, KB, DD, and AE performed the experiments; DB and JC performed the informatics analysis; AE, JC, CM, RG, DG, & KW wrote the manuscript. All authors read and approved the final manuscript.

## Pre-publication history

The pre-publication history for this paper can be accessed here:

http://www.biomedcentral.com/1755-8794/4/8/prepub

## Supplementary Material

Additional file 1 Supplemental information on methods, references for these methods, Table S1 (KEGG pathways enriched by differentially expressed genes or genes that are the presumptive targets of differentially expressed microRNAs) and Table S2 (Gene ontology terms associated with different network modules) Click here for file

Additional file 2 **Histochemical staining of lung samples**. Click here for file

Additional file 3 **Quantitative PCR verification of microarray data**. Click here for file

Additional file 4 **Top scoring gene and miRNA pairs which discriminate ILD from control and ILD subgroups**. Click here for file

Additional file 5 **Scatter plots of the most robust top scoring gene and miRNA pairs for various conditions**. Click here for file

Additional file 6 **Key pathways involved in the ILDs**. Click here for file

Additional file 7 **Modules of DEGs based on KEGG pathways**. Click here for file

Additional file 8 **Location of Zeb1 binding sites in the miR-23 distal promoter**. Click here for file
