# Supplementary material for: Systems biology of interstitial lung diseases: integration of mRNA and microRNA expression changes
Source: BMC Med Genomics. 2011 Jan 17;4:8. doi: 10.1186/1755-8794-4-8 (PMC3035594; doi:10.1186/1755-8794-4-8)
Supplement: Additional file 1 — Supplemental information on methods, references for these methods, Table S1 (KEGG pathways enriched by differentially expressed genes or genes that are the presumptive targets of differentially expressed microRNAs) and Table S2 (Gene ontology terms associated with different network modules) [file 1755-8794-4-8-S1.PDF]

Additional File 1

Supplemental Methods:

RNA isolation Total RNA was isolated from individual human lung samples by homogenization in TRIzol® Reagent per the manufacturer's protocol (Invitrogen, Carlsbad, CA) using Soft Tissue Omni Homogenizer Tips (Omni International; Marietta, GA). Following chloroform extraction, total RNA was precipitated with isopropanol, washed with ethanol, suspended in nuclease-free water, and stored at –70°C until used. Prior to cDNA synthesis, total RNA for each sample was evaluated for concentration and purity using a NanoDrop ND-1000 Spectrophotometer (NanoDrop; Wilmington, DE), and for integrity by gel electrophoresis using the FlashGel® RNA cassette system (Lonza; Rockland, ME).

Immunohistochemistry Lung tissue was fixed in neutral buffered formalin, sectioned, and stained with hematoxylin and eosin by standard methods as shown in Supplemental Figure 1. Photographs were taken using an Olympus 1X50 inverted microscope equipped with a 20x-objective and a Nikon camera (Olympus, Center Valley, PA) and the images were analyzed with analySIS GetIt software.

Messenger RNA and microRNA profiling with microarrays Total RNA (100 ng) was used to synthesize double-stranded cDNA using the Two-Cycle cDNA Synthesis Kit (Affymetrix, Santa Clara, CA). The cDNA served as a template to generate biotin-labeled antisense cRNA with an IVT Labeling Kit (Affymetrix). Labeled cRNA was fragmented and hybridized to the Affymetrix GeneChip® Human Genome U133 Plus 2.0 Array (containing 39,000 genes) as described in the Affymetrix GeneChip® protocol. Chip washing and staining were performed following the Affymetrix recommended protocol in Affymetrix Fluidics Station 450. The chips were scanned using an Affymetrix GeneChip® Scanner 3000 7G, and each digitized image was processed using the GeneChip® Operating Software (GCOS) version 1.2 (Affymetrix).

MicroRNAs were profiled by using microarrays from Agilent Technologies (G4470B, Santa Clara, CA) as well as by RT-PCR. The RNA samples were labeled and

processed as recommended by the manufacturer. Briefly, 100 ng of total RNA was dephosphorylated with calf intestinal alkaline phosphatase, followed by denaturation in dimethyl sulfoxide. T4 ligase was used to join cyanine 3-cytidine bisphosphate to the termini of all single-stranded RNA (including miRNA) and the labeled RNA was purified from these reactions by gel filtration on MicroBioSpin 6 columns (Bio-Rad). The purified labeled miRNA probes were hybridized for 20 h at 55 °C. The arrays were washed and scanned at 5-micron resolution with a PerkinElmer ScanArray Express array scanner. The resulting images were quantified by using Agilent's Feature Extraction software.

Real-time quantitative RT-PCR analysis The levels of selected mRNA and miRNA observed on microarray studies were validated by using a SYBR-based quantitative RT-PCR method [1]. Primers for selected miRNAs were obtained from Qiagen (Valencia CA). First strand cDNA to be used as a template for amplification was made from 50 ng of total RNA with a kit from Qiagen. About 2.5 ng of cDNA was then used in each amplification reactions with the Applied Biosystems 7900HT system. The amplification results were analyzed by SDS 2.2.2 software.

Identification of differentially expressed genes Affymetrix microarray data was preprocessed using GCRMA, a bioconductor package, for summarization, normalization and log2-transformation. To determine present and absent probesets, we used Gaussian mixture model and fitted two Gaussian probability density functions (PDFs), one for absent and the other for present probesets, to the normalized data. From 54675 probesets, 32746 probesets whose average intensity across all samples were larger than threshold value where two Gaussian PDFs met were declared to be present. These present probesets were further used for subsequent statistical testing in order to increase the ratio of true positives to false positives [2]. LIMMA, a bioconductor package, was used to identify differentially expressed genes (DEGs) between control and ILD patient samples. We defined DEGs as having positive false discovery rate [3] less than 0.1 (pFDR<0.1) and at least ±1.5-fold change between groups. To reduce redundancy of probesets mapped to the same Entrez ID, the single probeset showing the smallest pFDR value or largest average

intensity across all samples when more than one probeset had the same pFDR value was retained. As a result, 1423 DEGs uniquely mapped to Entrez IDs were identified.

For miRNA data from Agilent microarray, we first consolidated intensities of replicated probes with the identical sequence by taking median intensity and normalized the consolidated data using quantile method [4]. Present/absent calling of probes was performed as the same as mRNAs. Since small portion of miRNAs were identified as present, all probes were used for statistical testing. Similar to mRNAs, LIMMA was applied to find differentially expressed miRNAs (DEmiRNAs) between two groups and 125 present miRNAs showed pFDR<0.05 and at least ±1.5-fold changes were identified as DEmiRNAs.

Top scoring pair analysis The top scoring pair analysis (TSP) [5] was used to identify a pair of differentially expressed genes or miRNAs to discriminate ILD and control or subgroups of ILD samples. We performed TSP analysis based on all expressed genes and miRNAs using the software downloaded from http://ccbm.jhu.edu. TSP that appeared most frequently during leave-one-out cross-validation were retained.

Prediction of transcriptional regulatory interactions

In order to investigate transcriptional regulatory effect on DEGs, we used conserved transcription factor binding site (TFBS) information defined in 'tfbsConsSites' table of the UCSC genome database (hg18 assembly). In the table, chromosomal locations of 258 position-weight matrices (PWMs) representing TFBSs conserved in human, mouse and rat from TRANSFAC [6] were listed. We also retrieved gene coordinate information of 1423 DEGs from UCSC hg18 using 'RefGene' table and scanned the regulatory region of each DEG, from 5' end to 1kb upstream of 5' end, to find known binding sites of differentially expressed TFs (DETFs) in the region. A PWM can be found several times in the regulatory region of a DEG and multiple PWMs can be mapped to multiple DETFs. For example, two PWMs, V$ARNT_01 and V$ARNT_02, correspond to the same DETF gene, ARNT (conversion of PWM to TF gene was performed using index files provided with TRANSFAC). We simply defined the link between a DETF and a target DEG if any PWMs representing the DETF were found in the regulatory region of the DEG. As a

result, 1427 DETF-DEG pairs with 22 DETFs (i.e. well-characterized by UCSC database) and 598 target DEGs were identified. To find highly correlated DETF-DEG pairs, correlation coefficient between expression profile of DETF and DEG for each pair was computed ($\rho_{i,DETF-DEG}$, i = 1, 2, …, 1427). Next, we computed null correlations by randomly shuffling sample labels for expression profile of DETF and DEG, respectively ($\rho^{0}_{i,DETF-DEG}$, i = 1, 2, …, 1427). By 100 permutations of sample labels, we could get a set of null correlations, $\rho^{0b}_{1,DETF-DEG},\ldots,\rho^{0b}_{1427,DETF-DEG}$ (b = 1, 2, …, 100). P-value for each DETF-DEG pairs was computed by

$$p_{i,DETF-DEG} = \sum_{b=1}^{100} \frac{\#\left\{j : \left|\rho^{0b}_{j,DETF-DEG}\right| \geq \left|\rho_{i,DETF-DEG}\right|, j = 1,\ldots,1427\right\}}{1427 \cdot 100}, \ i = 1, 2, \ldots, 1427$$

Since we do not know exactly whether DETFs may act as activator or repressor, we accepted both positively and negatively correlated targets. False discovery rate ($FDR_{DETF-DEG}$) was obtained using Benjamini-Hochberg (BH) correction scheme [7].

The same procedure as above, except the definition of regulatory region of DEmiRNAs, was used to find DETF-DEmiRNA pairs. For 125 DEmiRNAs, their corresponding pre-miRNAs were identified using miRBase [8] and genomic coordinate information of these pre-miRNAs was downloaded from UCSC hg18 using ‘wgRNA’ table. Up to 10kb upstream region from 5’ end of pre-miRNA was used as the regulatory region. Finally, we obtained 541 DETF-DEmiRNA pairs and computed p-values ($p_{i,DETF-DEmiRNA}$, i = 1, 2, …, 541) and $FDR_{DETF-DEmiRNA}$ of them.

Prediction of miRNA-induced regulatory interactions TargetScanHuman V5.1 [9] was used to find potential target genes of miRNAs. Fist, we collected all predicted target genes of 125 DEmiRNAs and found 51901 DEmiRNA-target pairs containing 116 DEmiRNAs and 7547 target genes in human genome. Next, we compared the 7547 target genes with our 1423 DEGs and identified 6555 DEmiRNA-DEG pairs with 114 DEmiRNAs and 805 target DEGs. Also, we computed expression correlations of 6555 pairs based on the profiles of samples commonly used in both mRNA and miRNA microarray experiments. Null correlations were generated as the same as above. P-value ($p_{i,DEmiRNA-DEG}$, i = 1, 2, …, 6555) for each DEmiRNA-DEG pair was obtained by only

considering negative correlations (i.e. one-tailed manner) since miRNAs were believed to be negative regulators. P-values were further adjusted to $FDR_{DEmiRNA\text{-}DEG}$ using BH correction scheme.

Network generation and modularization To generate a network represents global changes of DEGs and the effect of key regulators, we used human protein-protein interaction (PPI) data downloaded from NCBI repository (September, 2009) and interactions obtained from KEGG database ('PathwayElementRelations' for all pathways were parsed using KEGG API in December, 2009). 374 PPIs and 554 KEGG interactions (without considering the direction of interactions) were derived from 480 DEGs out of 1423 (for brevity, self-interactions were removed). Also, we chose 436 DETF-DEG pairs (22 DETFs targeting 598 DEGs) having $FDR_{DETF\text{-}DEG}$ less than 0.05 as putative transcriptional interactions (we did not consider the direction of interactions). Taken together, the global network was generated using 640 DEGs and 1321 interactions. Network modularization techniques have been successfully used to decompose a large network into several modules in which nodes are highly inter-connected. We applied generalized topological overlap measure (GTOM, m=2) proposed by Yip and Horvath [10] to our network and identified 7 modules using hierarchical clustering of overlap matrix. Moreover, in order to investigate regulatory effect among regulators such as DETFs and DEmiRNAs, 55 DETF-DEmiRNA pairs (7 DETFs targeting 41 DEmiRNAs) showing $FDR_{DETF\text{-}DEmiRNA}$ less than 0.05 and 15 DEmiRNA-DETF pairs (13 DEmiRNAs targeting 6 DETFs) showing $FDR_{DEmiRNA\text{-}DEG}$ less than 0.1 were added to the network. As a result, a network contained 689 nodes (640 DEGs (including 22 well-characterized DETFs) and 49 DEmiRNAs) and 1391 non-redundant interactions. Although there were many significant DEmiRNA-DEG pairs (681 pairs having FDR<0.05 and 1470 pairs having FDR<0.1), we did not include them so that we could focus on the effects of DEmiRNA and DETF interactions. Network was constructed using Cytoscape [11].

Identification of putative feedforward loops (FFLs) composed of DETFs and DEmiRNAs A feedforward loop is composed of three interactions; (1) regulatory interaction from DETF to DEG, (2) from DETF to DEmiRNA and (3) from DEmiRNA

to DEG. For the first two interactions, statistically significant DETF-DEG pairs and DETF-DEmiRNA pairs were screened by the criterion, $FDR_{DETF\text{-}DEG}<0.1$ and $FDR_{DETF\text{-}DEmiRNA}<0.1$, respectively. Since DEmiRNA and target DEG (targeted by a DETF as well) may not be well correlated due to the incoherency in the FFL, we retained the result from TargetScan (i.e. 6555 DEmiRNA-DEG pairs) without any statistical filter for the third interaction. As a result, 111 DEGs putatively targeted by both DETF(s) and DEmiRNA(s) simultaneously and their potential regulators (13 well-characterized DETFs and 46 DEmiRNAs) were found to generate coherent and incoherent FFLs.

MYOCD/ZEB1 subgroups based on gene expression level Using individual gene expression profile of MYOCD (ZEB1), we computed mean (μ) and standard deviation (σ) of expression values in patient samples. Then, patient samples were divided into three groups; (1) low expression level; samples having expression value smaller than μ-σ, (2) medium expression level; samples with expression between μ-σ and μ+σ and (3) high expression level; samples having expression value larger than μ+σ. For MYOCD (ZEB1), 3 (3), 16 (15) and 4 (5) patient samples were assigned to low, medium and high expression level, respectively.

Supplemental References

1. Wang K, Zhang S, Marzolf B, Troisch P, Brightman A, Hu Z, Hood LE, Galas DJ: **Circulating microRNAs, potential biomarkers for drug-induced liver injury.** *Proc Natl Acad Sci U S A* 2009, **106:**4402-4407.
2. McClintick JN, Edenberg HJ: **Effects of filtering by Present call on analysis of microarray experiments.** *BMC Bioinformatics* 2006, **7:**49.
3. Storey JD, Tibshirani R: **Statistical significance for genomewide studies.** *Proc Natl Acad Sci U S A* 2003, **100:**9440-9445.
4. Bolstad BM, Irizarry RA, Astrand M, Speed TP: **A comparison of normalization methods for high density oligonucleotide array data based on variance and bias.** *Bioinformatics* 2003, **19:**185-193.
5. Tan AC, Naiman DQ, Xu L, Winslow RL, Geman D: **Simple decision rules for classifying human cancers from gene expression profiles.** *Bioinformatics* 2005, **21:**3896-3904.
6. Matys V, Fricke E, Geffers R, Gossling E, Haubrock M, Hehl R, Hornischer K, Karas D, Kel AE, Kel-Margoulis OV, et al: **TRANSFAC: transcriptional regulation, from patterns to profiles.** *Nucleic Acids Res* 2003, **31:**374-378.

7. Benjamini Y, Hochberg Y: **Controlling the false discovery rate: a practical and powerful approach to multiple testing.** *J Royal Statistical Society, Series B* 1995, **57:**289-300.
8. Griffiths-Jones S, Grocock RJ, van Dongen S, Bateman A, Enright AJ: **miRBase: microRNA sequences, targets and gene nomenclature.** *Nucleic Acids Res* 2006, **34:**D140-144.
9. Lewis BP, Burge CB, Bartel DP: **Conserved seed pairing, often flanked by adenosines, indicates that thousands of human genes are microRNA targets.** *Cell* 2005, **120:**15-20.
10. Yip AM, Horvath S: **Gene network interconnectedness and the generalized topological overlap measure.** *BMC Bioinformatics* 2007, **8:**22.
11. Shannon P, Markiel A, Ozier O, Baliga NS, Wang JT, Ramage D, Amin N, Schwikowski B, Ideker T: **Cytoscape: a software environment for integrated models of biomolecular interaction networks.** *Genome Res* 2003, **13:**2498-2504.

Table S1. KEGG pathways enriched by DEGs, all DEmiRNA target genes and all DEmiRNA target DEGs using one-sided Fisher exact test.

KEGG Pathway			Up-regulated DEGs	Down-regulated DEGs	All targets of down-regulated DEmiRNAs	All targets of up-regulated DEmiRNAs	DEG targets of down-regulated DEmiRNAs	DEG targets of up-regulated DEmiRNAs
Metabolism	Amino Acid Metabolism	Cysteine and methionine metabolism		6.3E-2				
		Glycine, serine and threonine metabolism		**9.6E-3**				
		Lysine degradation	6.8E-2					
		Valine, leucine and isoleucine degradation		1.3E-2				
	Biosynthesis of Secondary Metabolites	Terpenoid backbone biosynthesis		2.7E-2				2.4E-2
	Carbohydrate Metabolism	Amino sugar and nucleotide sugar metabolism				4.1E-2		
		Ascorbate and aldarate metabolism			4.9E-2			
		Citrate cycle (TCA cycle)						9.7E-2
		Inositol phosphate metabolism			3.3E-2	2.5E-2		
		Pentose and glucuronate interconversions			1.8E-2			
	Energy Metabolism	Reductive carboxylate cycle (CO_2 fixation)	4.2E-2					
	Glycan Biosynthesis and Metabolism	Chondroitin sulfate biosynthesis				2.3E-2		
		Glycosaminoglycan degradation	2.4E-2					
		Glycosphingolipid biosynthesis				2.0E-2		
		Heparan sulfate biosynthesis			2.5E-2	**2.2E-3**		
		Keratan sulfate biosynthesis				1.6E-2		
		N-Glycan biosynthesis				3.7E-2		
		O-Glycan biosynthesis	8.5E-2		7.9E-2	1.0E-2		
	Lipid Metabolism	Biosynthesis of unsaturated fatty acids				9.4E-2		
		Fatty acid biosynthesis						9.5E-2
		Fatty acid elongation in mitochondria	2.8E-2				5.7E-2	
		Fatty acid metabolism						3.1E-2
		Glycerolipid metabolism				9.5E-2		3.7E-2
		Glycerophospholipid metabolism			5.9E-2	2.1E-2		
		Sphingolipid metabolism				1.1E-2		
		Steroid biosynthesis		**5.8E-3**				
		Synthesis and degradation of ketone bodies		5.8E-2				
	Metabolism of Cofactors and Vitamins	Nicotinate and nicotinamide metabolism		2.0E-2				5.8E-2
		Thiamine metabolism				1.1E-2		
	Metabolism of Other Amino Acids	D-Glutamine and D-glutamate metabolism			2.8E-2	1.7E-2		
		Selenoamino acid metabolism		2.7E-2				
	Xenobiotics Biodegradation and Metabolism	Caprolactam degradation	1.1E-2					
Genetic Information Processing	Folding, Sorting and Degradation	RNA degradation		1.6E-2				
		SNARE interactions in vesicular transport			6.6E-2	**1.8E-3**		
		Ubiquitin mediated proteolysis			**3.0E-4**	***4.1E-7***		7.4E-2
Environmental Information Processing	Signal Transduction	Calcium signaling pathway			**4.6E-4**	**2.7E-3**	4.1E-2	
		ErbB signaling pathway			***9.3E-5***	***6.4E-8***		

		Hedgehog signaling pathway	4.2E-2		2.8E-2	**6.2E-4**	**8.1E-3**	
		Jak-STAT signaling pathway			7.6E-2	7.6E-2		
		MAPK signaling pathway		9.5E-2	***1.7E-8***	***1.3E-16***		
		mTOR signaling pathway			1.1E-2	***2.6E-7***		5.3E-2
		Notch signaling pathway	7.2E-2			**5.4E-3**		
		Phosphatidylinositol signaling system			***8.2E-5***	***3.9E-6***		
		TGF-beta signaling pathway			***4.3E-8***	***1.9E-6***		
		VEGF signaling pathway			7.4E-2	**2.2E-3**		3.6E-2
		Wnt signaling pathway	**4.9E-3**	**7.4E-3**	***1.4E-5***	***1.3E-14***	**7.2E-4**	**3.3E-3**
	Signaling Molecules and Interaction	Cell adhesion molecules (CAMs)	8.1E-2				7.4E-2	
		ECM-receptor interaction	***5.7E-7***		**2.7E-4**	***9.3E-6***	**3.1E-3**	
Cellular Processes	Behavior	Circadian rhythm - mammal			3.1E-2	1.6E-2		
	Cell Communication	Adherens junction			**1.1E-4**	***1.1E-9***		
		Focal adhesion	***9.6E-9***		***1.4E-7***	***8.8E-17***	**3.1E-3**	
		Gap junction			1.1E-2	***4.7E-6***	2.8E-2	
		Tight junction			**8.3E-3**	***3.9E-6***		
	Cell Growth and Death	Apoptosis		4.4E-2	2.7E-2	**6.5E-3**		
		Cell cycle			**7.3E-3**			
		p53 signaling pathway		8.6E-2	1.5E-2	**4.4E-3**		
	Cell Motility	Regulation of actin cytoskeleton			***2.2E-5***	***1.4E-12***		
	Circulatory System	Vascular smooth muscle contraction	***5.6E-6***		**3.0E-3**	**1.0E-3**	**1.6E-4**	
	Development	Axon guidance	6.8E-2		***1.2E-8***	***3.9E-15***	6.7E-2	
		Dorso-ventral axis formation			**4.0E-4**	1.3E-2		
	Endocrine System	Adipocytokine signaling pathway		**1.5E-4**	2.0E-2	***2.3E-5***		**7.4E-4**
		GnRH signaling pathway			**4.8E-3**	**1.6E-4**	3.8E-2	
		Insulin signaling pathway	**2.2E-3**	1.9E-2	**2.6E-3**	***7.0E-11***	7.9E-2	2.5E-2
		Melanogenesis			**2.9E-3**	***1.2E-6***		
		PPAR signaling pathway						2.6E-2
	Immune System	B cell receptor signaling pathway	1.3E-2		3.8E-2	**6.6E-3**		
		Chemokine signaling pathway			4.1E-2	**7.3E-3**		
		Complement and coagulation cascades		3.3E-2				
		Fc epsilon RI signaling pathway			1.1E-2	**2.8E-3**		
		Fc gamma R-mediated phagocytosis			**1.9E-4**	***5.2E-7***		
		Leukocyte transendothelial migration				**8.7E-4**		
		T cell receptor signaling pathway			**4.1E-4**	***1.7E-6***		
		Toll-like receptor signaling pathway		1.4E-2				
	Nervous System	Long-term depression			1.2E-2	5.6E-2	9.7E-2	
		Long-term potentiation			***7.3E-7***	***4.0E-7***	1.5E-2	
		Neurotrophin signaling pathway		5.6E-2	***4.1E-7***	***3.6E-15***		
	Transport and Catabolism	Endocytosis			***7.8E-6***	***2.1E-14***		3.3E-2
		Lysosome	**5.1E-3**			1.7E-2		
Human Diseases	Cancers	Acute myeloid leukemia			2.4E-2	***4.6E-6***		
		Basal cell carcinoma	**9.9E-3**		4.0E-2	***8.5E-6***	**7.3E-3**	
		Bladder cancer				2.2E-2		
		Chronic myeloid leukemia	4.0E-2		***7.2E-6***	***2.2E-7***	**2.0E-3**	
		Colorectal cancer			***1.5E-5***	***2.6E-9***		
		Endometrial cancer			1.1E-2	**1.6E-4**		
		Glioma			***2.2E-5***	***5.0E-8***		
		Melanoma			**9.4E-4**	***7.4E-6***		
		Non-small cell lung cancer			**1.2E-3**	***1.6E-5***		
		Pancreatic cancer			**1.2E-3**	***1.2E-5***		

	Pathways in cancer	**3.4E-3**	9.9E-2	***2.9E-9***	***3.1E-19***	**4.8E-4**	
	Prostate cancer			**1.6E-4**	***3.8E-10***		
	Renal cell carcinoma	8.6E-2		**1.1E-4**	***3.4E-10***		
	Small cell lung cancer		8.8E-2	**4.4E-4**	***2.3E-5***		
	Thyroid cancer				1.1E-2		
Circulatory Diseases	Arrhythmogenic right ventricular cardiomyopathy	**9.2E-4**		**2.9E-3**	**4.7E-4**		
	Hypertrophic cardiomyopathy (HCM)	**5.1E-4**		**7.7E-3**	**4.2E-3**		
Infectious Diseases	Epithelial cell signaling in H. pylori infection		***6.9E-6***		**3.2E-3**		2.5E-2
Metabolic Disorders	Maturity onset diabetes of the young			1.8E-2			
	Type II diabetes mellitus			**3.2E-3**	**5.4E-3**		
Neurodegenerative Diseases	Alzheimer's disease					3.5E-2	
	Amyotrophic lateral sclerosis (ALS)			***6.5E-5***	**2.5E-4**		6.1E-2

The significance level of the calculated p-value is represented by regular (0.01~0.1), bold (0.0001~0.01) and bold-Italic (<0.0001) fonts.

Table S2. Gene ontology (GO) terms associated with different network modules.

			Module						
GO ID	GO Description	Class	1	2	3	4	5	6	7
GO:0006732	coenzyme metabolic process	BP	***1.0E-4***						
GO:0051186	cofactor metabolic process	BP	**6.0E-3**						
GO:0009058	biosynthetic process	BP	**9.0E-3**						
GO:0044237	cellular metabolic process	BP	1.3E-2						
GO:0032787	monocarboxylic acid metabolic process	BP	1.5E-2						
GO:0044238	primary metabolic process	BP	1.5E-2						
GO:0006575	cellular amino acid derivative metabolic process	BP	1.8E-2						
GO:0006790	sulfur metabolic process	BP	1.8E-2						
GO:0016054	organic acid catabolic process	BP	2.1E-2						
GO:0046395	carboxylic acid catabolic process	BP	2.1E-2						
GO:0006631	fatty acid metabolic process	BP	3.7E-2						
GO:0008152	metabolic process	BP	3.9E-2						
GO:0019752	carboxylic acid metabolic process	BP	4.6E-2						
GO:0043436	oxoacid metabolic process	BP	4.6E-2						
GO:0016053	organic acid biosynthetic process	BP	4.9E-2						
GO:0046394	carboxylic acid biosynthetic process	BP	4.9E-2						
GO:0006082	organic acid metabolic process	BP	4.9E-2						
GO:0042180	cellular ketone metabolic process	BP	5.5E-2						
GO:0006749	glutathione metabolic process	BP	5.5E-2						
GO:0044249	cellular biosynthetic process	BP	5.6E-2						
GO:0006519	cellular amino acid and derivative metabolic process	BP	6.9E-2						
GO:0003012	muscle system process	BP	8.0E-2						
GO:0042221	response to chemical stimulus	BP		***1.0E-4***					2.9E-2
GO:0048519	negative regulation of biological process	BP		***1.0E-4***					
GO:0007165	signal transduction	BP		***1.0E-4***					
GO:0048523	negative regulation of cellular process	BP		***1.0E-4***					
GO:0007154	cell communication	BP		***1.0E-4***					
GO:0048522	positive regulation of cellular process	BP		***1.0E-4***					
GO:0050793	regulation of developmental process	BP		***1.0E-4***					
GO:0048518	positive regulation of biological process	BP		***1.0E-4***					
GO:0050794	regulation of cellular process	BP		***1.0E-4***					
GO:0042127	regulation of cell proliferation	BP		***1.0E-4***					
GO:0009987	cellular process	BP		***1.0E-4***					
GO:0065007	biological regulation	BP		***1.0E-4***					
GO:0042981	regulation of apoptosis	BP		***1.0E-4***					
GO:0043067	regulation of programmed cell death	BP		***1.0E-4***					
GO:0010941	regulation of cell death	BP		***1.0E-4***					
GO:0043065	positive regulation of apoptosis	BP		***1.0E-4***					
GO:0043068	positive regulation of programmed cell death	BP		***1.0E-4***					
GO:0010942	positive regulation of cell death	BP		***1.0E-4***					
GO:0006464	protein modification process	BP		***1.0E-4***					
GO:0006793	phosphorus metabolic process	BP		***1.0E-4***					
GO:0006796	phosphate metabolic process	BP		***1.0E-4***					
GO:0006935	chemotaxis	BP		***1.0E-4***					
GO:0007166	cell surface receptor linked signal transduction	BP		***1.0E-4***					
GO:0007167	enzyme linked receptor protein signaling pathway	BP		***1.0E-4***					
GO:0007169	transmembrane receptor protein tyrosine kinase signaling pathway	BP		***1.0E-4***					
GO:0007242	intracellular signaling cascade	BP		***1.0E-4***					
GO:0007610	behavior	BP		***1.0E-4***					
GO:0007626	locomotory behavior	BP		***1.0E-4***					
GO:0008150	biological_process	BP		***1.0E-4***					
GO:0008284	positive regulation of cell proliferation	BP		***1.0E-4***					
GO:0009966	regulation of signal transduction	BP		***1.0E-4***					
GO:0009968	negative regulation of signal transduction	BP		***1.0E-4***					
GO:0010646	regulation of cell communication	BP		***1.0E-4***					
GO:0010648	negative regulation of cell communication	BP		***1.0E-4***					
GO:0016310	phosphorylation	BP		***1.0E-4***					
GO:0030334	regulation of cell migration	BP		***1.0E-4***					
GO:0030335	positive regulation of cell migration	BP		***1.0E-4***					
GO:0032879	regulation of localization	BP		***1.0E-4***					
GO:0040011	locomotion	BP		***1.0E-4***					
GO:0040012	regulation of locomotion	BP		***1.0E-4***					
GO:0040017	positive regulation of locomotion	BP		***1.0E-4***					
GO:0042330	taxis	BP		***1.0E-4***					
GO:0043687	post-translational protein modification	BP		***1.0E-4***					
GO:0050789	regulation of biological process	BP		***1.0E-4***					
GO:0051270	regulation of cellular component movement	BP		***1.0E-4***					
GO:0051272	positive regulation of cellular component movement	BP		***1.0E-4***					
GO:0032000	positive regulation of fatty acid beta-oxidation	BP		**1.0E-3**					
GO:0043412	macromolecule modification	BP		**1.0E-3**					
GO:0032502	developmental process	BP		**2.0E-3**					
GO:0006468	protein amino acid phosphorylation	BP		**2.0E-3**					
GO:0048513	organ development	BP		**2.0E-3**					
GO:0050896	response to stimulus	BP		**6.0E-3**					
GO:0006915	apoptosis	BP		**6.0E-3**					
GO:0046626	regulation of insulin receptor signaling pathway	BP		**6.0E-3**					
GO:0051239	regulation of multicellular organismal process	BP		**6.0E-3**					
GO:0012501	programmed cell death	BP		1.1E-2					
GO:0051094	positive regulation of developmental process	BP		1.3E-2					
GO:0009953	dorsal/ventral pattern formation	BP		1.3E-2					
GO:0045597	positive regulation of cell differentiation	BP		1.7E-2					
GO:0031998	regulation of fatty acid beta-oxidation	BP		2.1E-2					
GO:0046321	positive regulation of fatty acid oxidation	BP		2.1E-2					
GO:0045595	regulation of cell differentiation	BP		2.3E-2					
GO:0031346	positive regulation of cell projection organization	BP		2.3E-2					
GO:0006928	cellular component movement	BP		2.7E-2					
GO:0007229	integrin-mediated signaling pathway	BP		2.7E-2					
GO:0070887	cellular response to chemical stimulus	BP		2.7E-2					
GO:0031274	positive regulation of pseudopodium assembly	BP		2.9E-2					
GO:0008219	cell death	BP		3.1E-2					
GO:0031344	regulation of cell projection organization	BP		3.2E-2					
GO:0007173	epidermal growth factor receptor signaling pathway	BP		3.2E-2					
GO:0016265	death	BP		3.2E-2					
GO:0030879	mammary gland development	BP		3.2E-2					
GO:0031272	regulation of pseudopodium assembly	BP		3.6E-2					
GO:0045725	positive regulation of glycogen biosynthetic process	BP		3.6E-2					
GO:0048583	regulation of response to stimulus	BP		3.6E-2					

GO:0010033	response to organic substance	BP		3.8E-2			1.4E-2
GO:0010819	regulation of T cell chemotaxis	BP		9.7E-2			
GO:0010820	positive regulation of T cell chemotaxis	BP		9.7E-2			
GO:0018347	protein amino acid farnesylation	BP		9.7E-2			
GO:0021775	smoothened signaling pathway involved in ventral spinal cord interneuron specification	BP		9.7E-2			
GO:0021776	smoothened signaling pathway involved in spinal cord motor neuron cell fate specification	BP		9.7E-2			
GO:0010556	regulation of macromolecule biosynthetic process	BP			4.7E-2		
GO:0031326	regulation of cellular biosynthetic process	BP			8.9E-2		
GO:0009889	regulation of biosynthetic process	BP			9.0E-2		
GO:0045683	negative regulation of epidermis development	BP				**4.0E-3**	
GO:0051257	spindle midzone assembly involved in meiosis	BP				**5.0E-3**	
GO:0051280	negative regulation of release of sequestered calcium ion into cytosol	BP				2.4E-2	
GO:0051453	regulation of intracellular pH	BP				3.3E-2	
GO:0051383	kinetochore organization	BP				3.5E-2	
GO:0051450	myoblast proliferation	BP				3.5E-2	
GO:0051384	response to glucocorticoid stimulus	BP				3.6E-2	
GO:0050918	positive chemotaxis	BP				3.7E-2	
GO:0044248	cellular catabolic process	BP				6.7E-2	
GO:0051533	positive regulation of NFAT protein import into nucleus	BP				7.1E-2	
GO:0051656	establishment of organelle localization	BP				7.1E-2	
GO:0048554	positive regulation of metalloenzyme activity	BP				7.3E-2	
GO:0046320	regulation of fatty acid oxidation	BP					***1.0E-4***
GO:0019217	regulation of fatty acid metabolic process	BP					**3.0E-3**
GO:0010565	regulation of cellular ketone metabolic process	BP					1.5E-2
GO:0006633	fatty acid biosynthetic process	BP					2.9E-2
GO:0034097	response to cytokine stimulus	BP					2.9E-2
GO:0019220	regulation of phosphate metabolic process	BP					4.1E-2
GO:0051174	regulation of phosphorus metabolic process	BP					4.1E-2
GO:0010562	positive regulation of phosphorus metabolic process	BP					7.0E-2
GO:0045937	positive regulation of phosphate metabolic process	BP					7.0E-2
GO:0019216	regulation of lipid metabolic process	BP					9.6E-2
GO:0044444	cytoplasmic part	CC	***1.0E-4***	**2.0E-3**			
GO:0044424	intracellular part	CC	***1.0E-4***				
GO:0005737	cytoplasm	CC	**5.0E-3**	1.3E-2			
GO:0044449	contractile fiber part	CC	4.5E-2				
GO:0005829	cytosol	CC		***1.0E-4***			
GO:0005886	plasma membrane	CC		***1.0E-4***			
GO:0044459	plasma membrane part	CC		**1.0E-3**			
GO:0005923	tight junction	CC		**2.0E-3**			
GO:0070160	occluding junction	CC		**2.0E-3**			
GO:0042995	cell projection	CC		2.7E-2			
GO:0031588	AMP-activated protein kinase complex	CC					***1.0E-4***
GO:0005515	protein binding	MF	***1.0E-4***	***1.0E-4***			
GO:0005516	calmodulin binding	MF	**5.0E-3**				
GO:0005488	binding	MF	4.7E-2	***1.0E-4***			
GO:0019904	protein domain specific binding	MF		***1.0E-4***			
GO:0004672	protein kinase activity	MF		***1.0E-4***			
GO:0004871	signal transducer activity	MF		***1.0E-4***			
GO:0016301	kinase activity	MF		***1.0E-4***			
GO:0016773	phosphotransferase activity, alcohol group as acceptor	MF		***1.0E-4***			
GO:0017124	SH3 domain binding	MF		***1.0E-4***			
GO:0060089	molecular transducer activity	MF		***1.0E-4***			
GO:0004714	transmembrane receptor protein tyrosine kinase activity	MF		**2.0E-3**			
GO:0032403	protein complex binding	MF		**6.0E-3**			
GO:0004713	protein tyrosine kinase activity	MF		1.3E-2			
GO:0005102	receptor binding	MF		1.3E-2			
GO:0016772	transferase activity, transferring phosphorus-containing groups	MF		1.3E-2			
GO:0019199	transmembrane receptor protein kinase activity	MF		1.3E-2			
GO:0004918	interleukin-8 receptor activity	MF		9.7E-2			
GO:0005158	insulin receptor binding	MF		9.7E-2			
GO:0051536	iron-sulfur cluster binding	MF				3.8E-2	
GO:0051540	metal cluster binding	MF				6.4E-2	
GO:0019901	protein kinase binding	MF					1.7E-2
GO:0019900	kinase binding	MF					2.9E-2
GO:0004860	protein kinase inhibitor activity	MF					7.2E-2
GO:0019210	kinase inhibitor activity	MF					9.5E-2

With genes in each module of the network, GO enrichment analysis was performed using FuncAssociate (http://llama.med.harvard.edu/funcassociate/). The significance level of the p-value adjusted by this method is represented by regular (0.01~0.1), bold (0.001~0.01), and bold-Italic (<0.001) fonts.
