# Supplementary material for: Systems biology of interstitial lung diseases: integration of mRNA and microRNA expression changes
Source: BMC Med Genomics. 2011 Jan 17;4:8. doi: 10.1186/1755-8794-4-8 (PMC3035594; doi:10.1186/1755-8794-4-8)

Additional file 2. Histochemical staining of lung samples. Panels A & B, normal lung; panels C&D, IPF lung with forced vital capacity score 1; panels E&F, IPF with forced vital capacity score 2. Panels A, C, & E were stained with hematoxylin and eosin. Panels B, D, & F were stained with Masson's Trichrome. Magnification 15x; calibration bar in panel E is xx microns. C & E show the replacement of regular alveolar structures with extensive extracellular matrix and heavy fibrosis which is characteristic of UIP/IPF. D & F show prominent staining for fibrillar collagen in the extracellular matrix. The scale bar in panel E represents 500 microns. Method: Lung tissue was fixed in neutral buffered formalin, sectioned, and stained with hematoxylin and eosin by standard methods. Photographs were taken using an Olympus 1X50 inverted microscope equipped with a 20x-objective and a Nikon camera (Olympus, Center Valley, PA) and the images were analyzed with analySIS GetIt software.

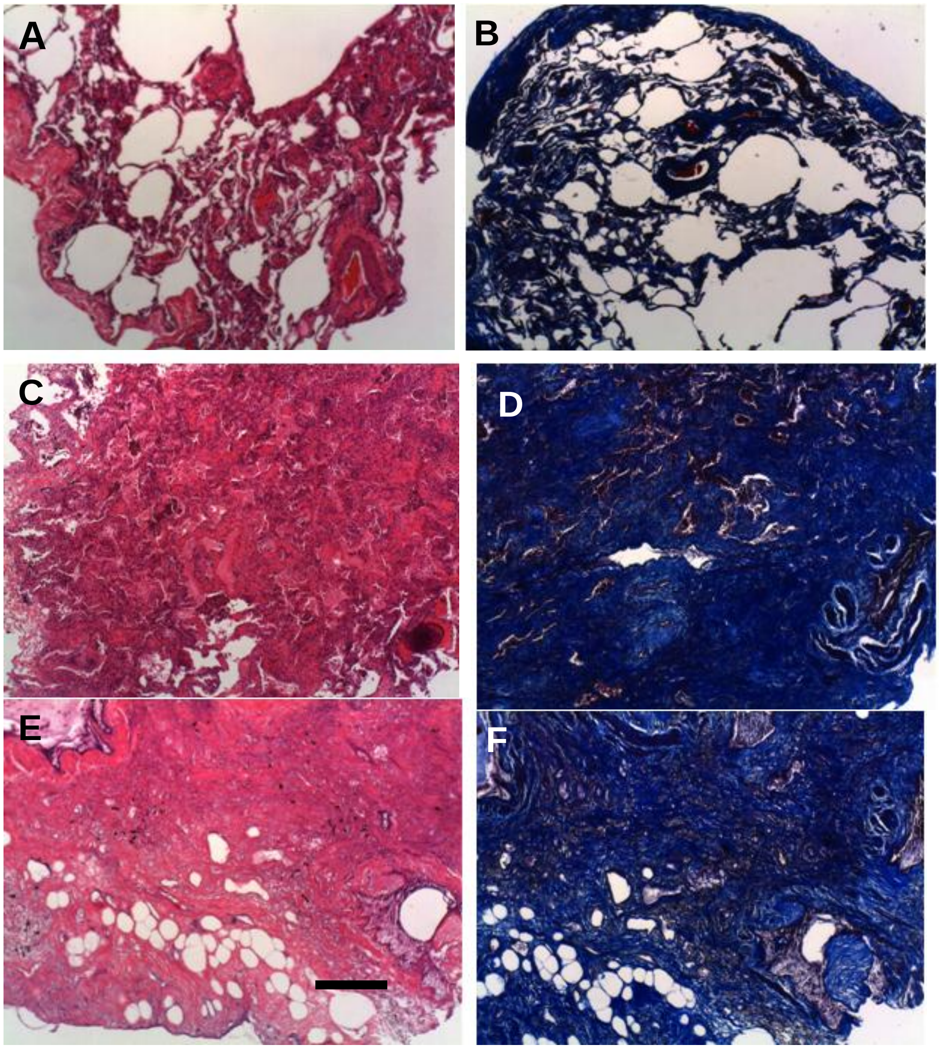

Supplement: Additional file 2 — Histochemical staining of lung samples. [file 1755-8794-4-8-S2.PDF]
