# Supplementary material for: Systems biology of interstitial lung diseases: integration of mRNA and microRNA expression changes
Source: BMC Med Genomics. 2011 Jan 17;4:8. doi: 10.1186/1755-8794-4-8 (PMC3035594; doi:10.1186/1755-8794-4-8)

Additional file 3. Results of qPCR verification of microarray data. The changes of selected genes were verified through qPCR. The qPCR data were presented as lines and the array data were shown as bars.

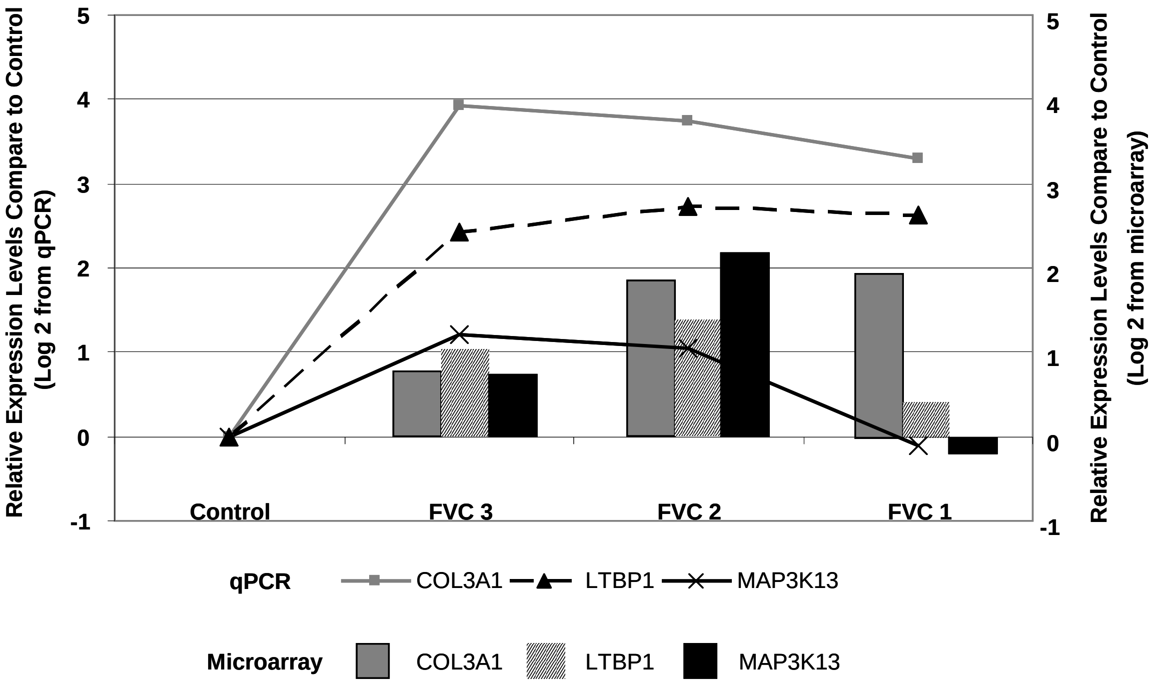

Supplement: Additional file 3 — Quantitative PCR verification of microarray data. [file 1755-8794-4-8-S3.PDF]
