# Supplementary material for: Systems biology of interstitial lung diseases: integration of mRNA and microRNA expression changes
Source: BMC Med Genomics. 2011 Jan 17;4:8. doi: 10.1186/1755-8794-4-8 (PMC3035594; doi:10.1186/1755-8794-4-8)

Additional file 4. Top scoring pair analysis. **A**. The 18 indicated DEGs resulted in the identification of 10 top scoring pairs that could discriminate control (black bar) from ILD (red bar). **B**. The 23 indicated DEGs resulted in the identification of 12 top scoring pairs that could discriminate ILD subgroup 1 profiles (black bar largely ILD excluding IPF) from subgroups 2 and 3 (red bar, largely UIP/IPF). **C**. The 12 indicated DEGs resulted in the identification of 6 top scoring pairs that could discriminate IPF with FVC 2+3 patients (black bar) from IPF with FVC 1 (red bar). **D.** The 18 differentially expressed miRNAs yielded 9 top scoring pairs that could discriminate ILD (red bar) from control lung tissue (black bar).

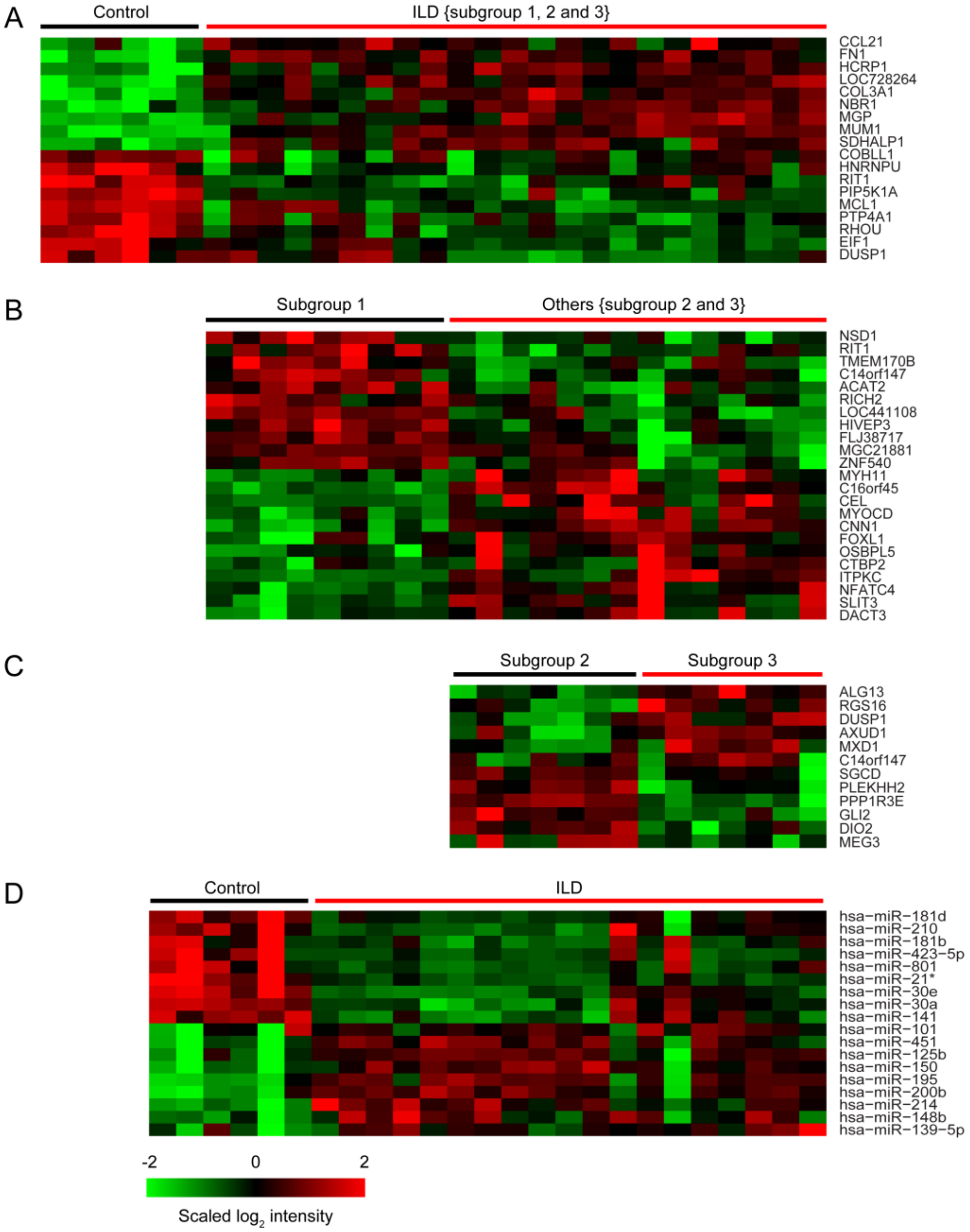

Supplement: Additional file 4 — Top scoring gene and miRNA pairs which discriminate ILD from control and ILD subgroups. [file 1755-8794-4-8-S4.PDF]
