# Supplementary material for: Systems biology of interstitial lung diseases: integration of mRNA and microRNA expression changes
Source: BMC Med Genomics. 2011 Jan 17;4:8. doi: 10.1186/1755-8794-4-8 (PMC3035594; doi:10.1186/1755-8794-4-8)

Additional file 5. Top scoring pair (TSP) analysis results. Scatter plots that present the most robust DEG pair (A) or miRNA pair (B) that can discriminate the ILD samples (filled circles) from controls (open circles) based on the results of TSP analyses on all present probesets and all samples. A DEG pair was identified that distinguishes patients with IPF (filled circles) from patients diagnosed with NSIP (open circles) (C).

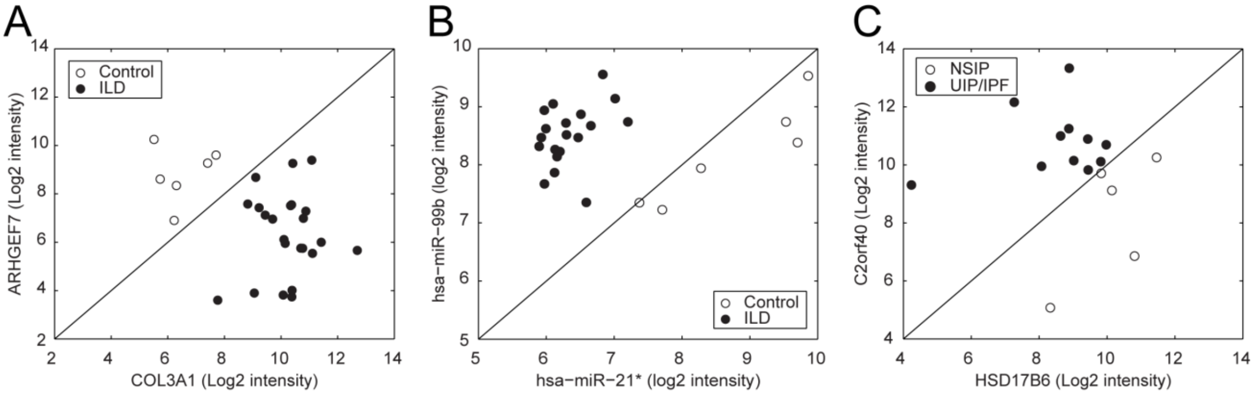

Supplement: Additional file 5 — Scatter plots of the most robust top scoring gene and miRNA pairs for various conditions. [file 1755-8794-4-8-S5.PDF]
