# Supplementary material for: Systems biology of interstitial lung diseases: integration of mRNA and microRNA expression changes
Source: BMC Med Genomics. 2011 Jan 17;4:8. doi: 10.1186/1755-8794-4-8 (PMC3035594; doi:10.1186/1755-8794-4-8)

Additional file 6. Key pathways involved in the ILDs. (A) The TGF-β pathway in ILD samples.

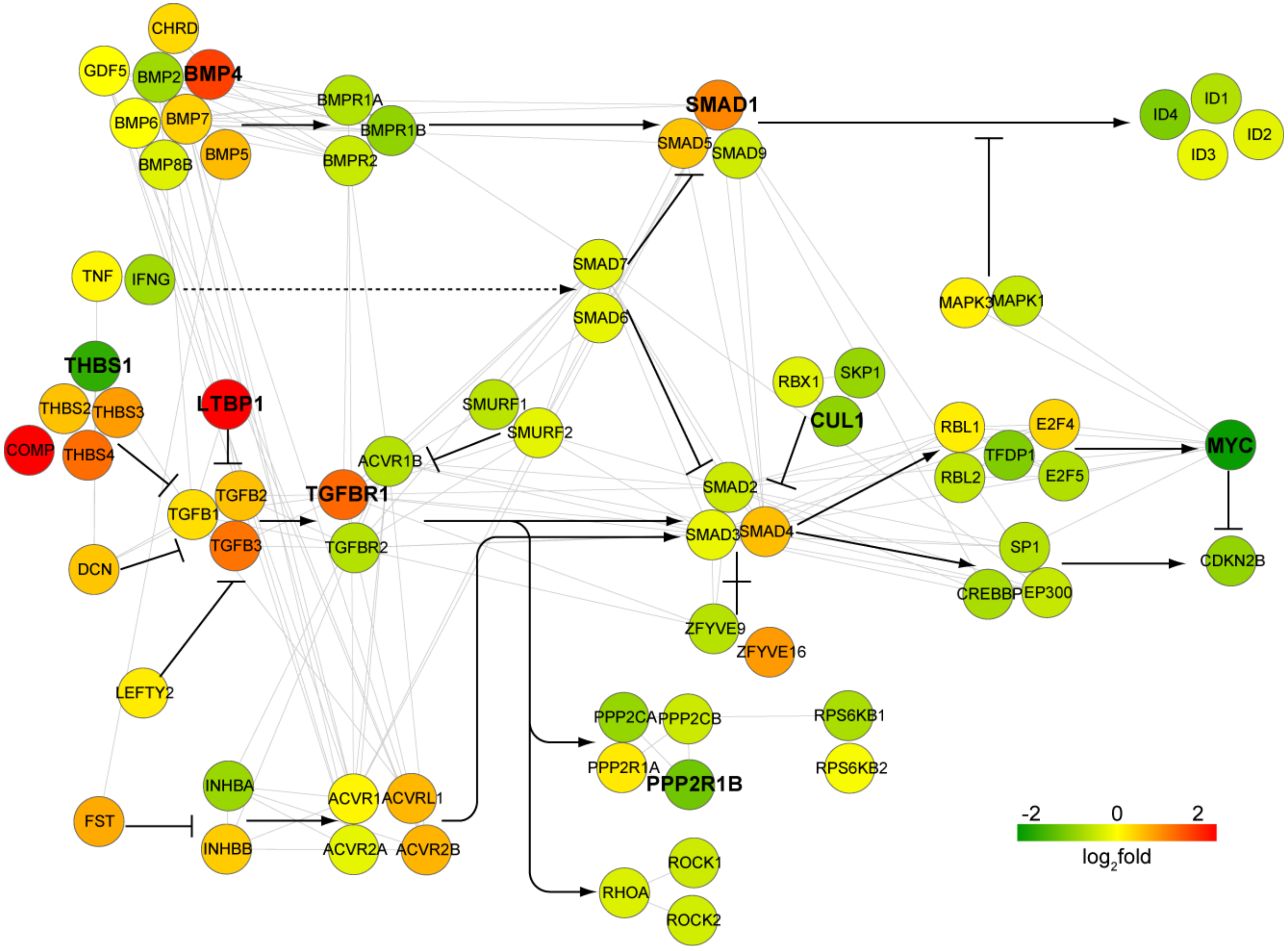

(B) The Toll like receptor signaling pathway in ILD samples.

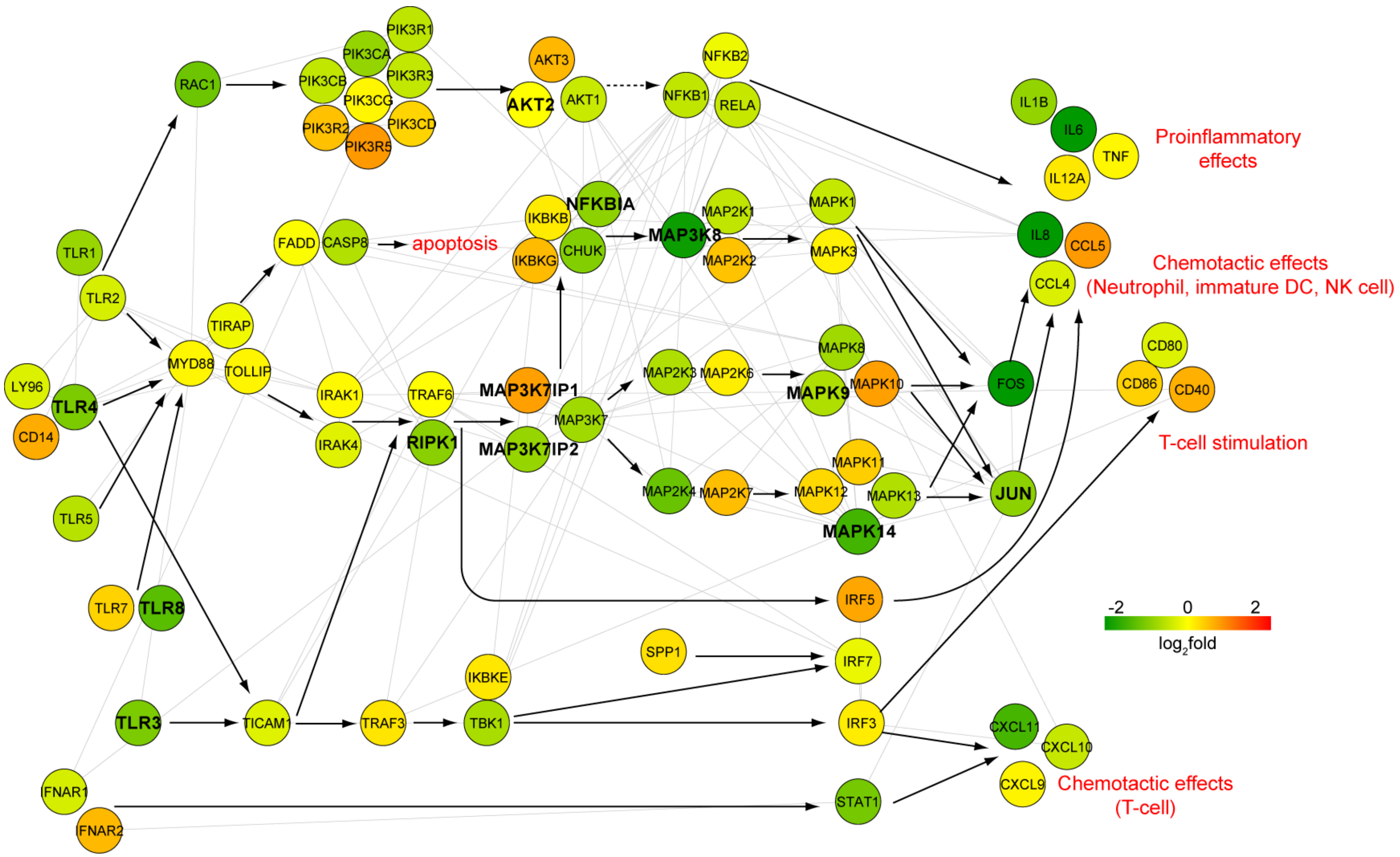

(C) The Insulin signaling pathway in ILD samples.

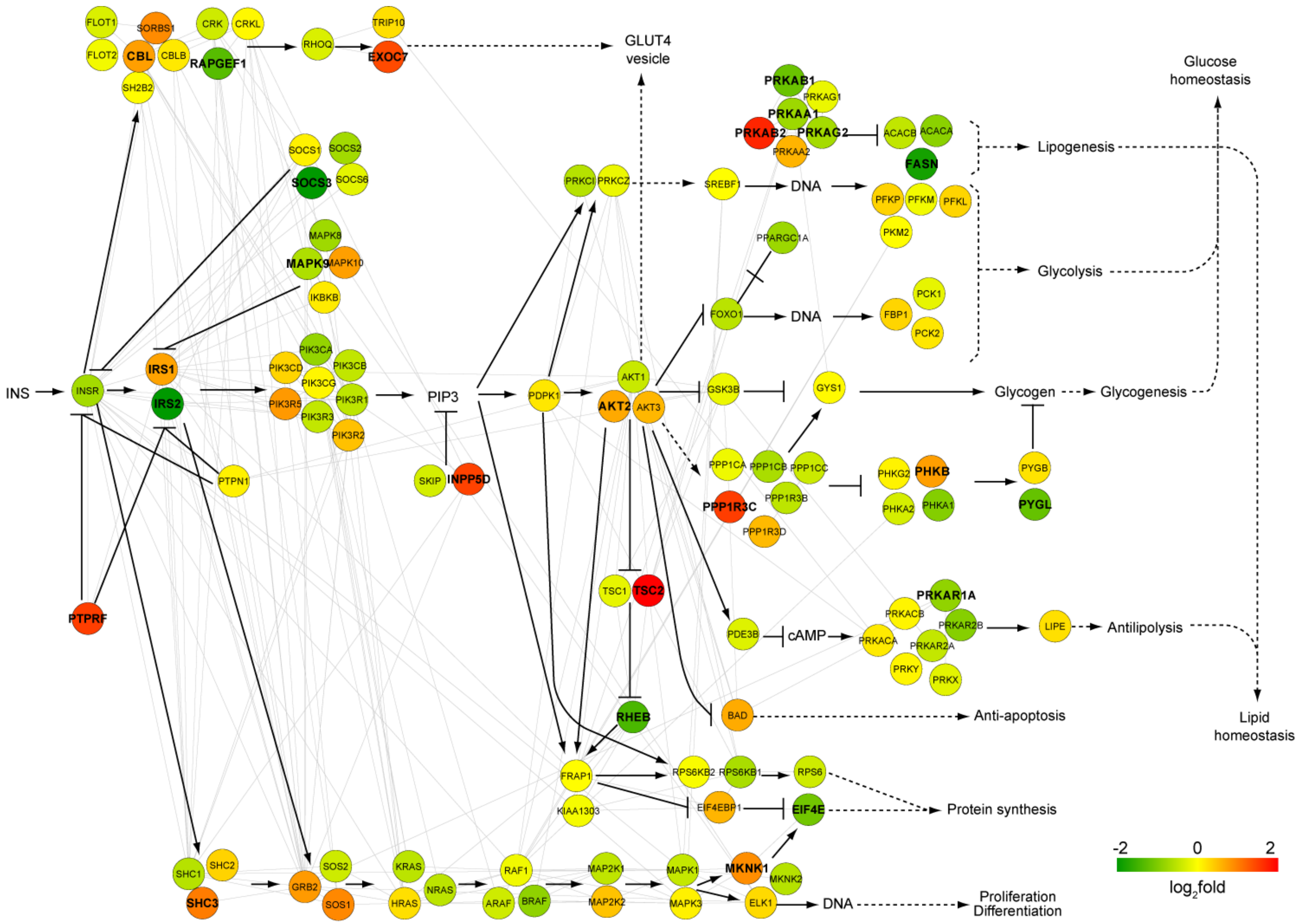

(D) Alpha-smooth muscle actin signaling network in ILD.

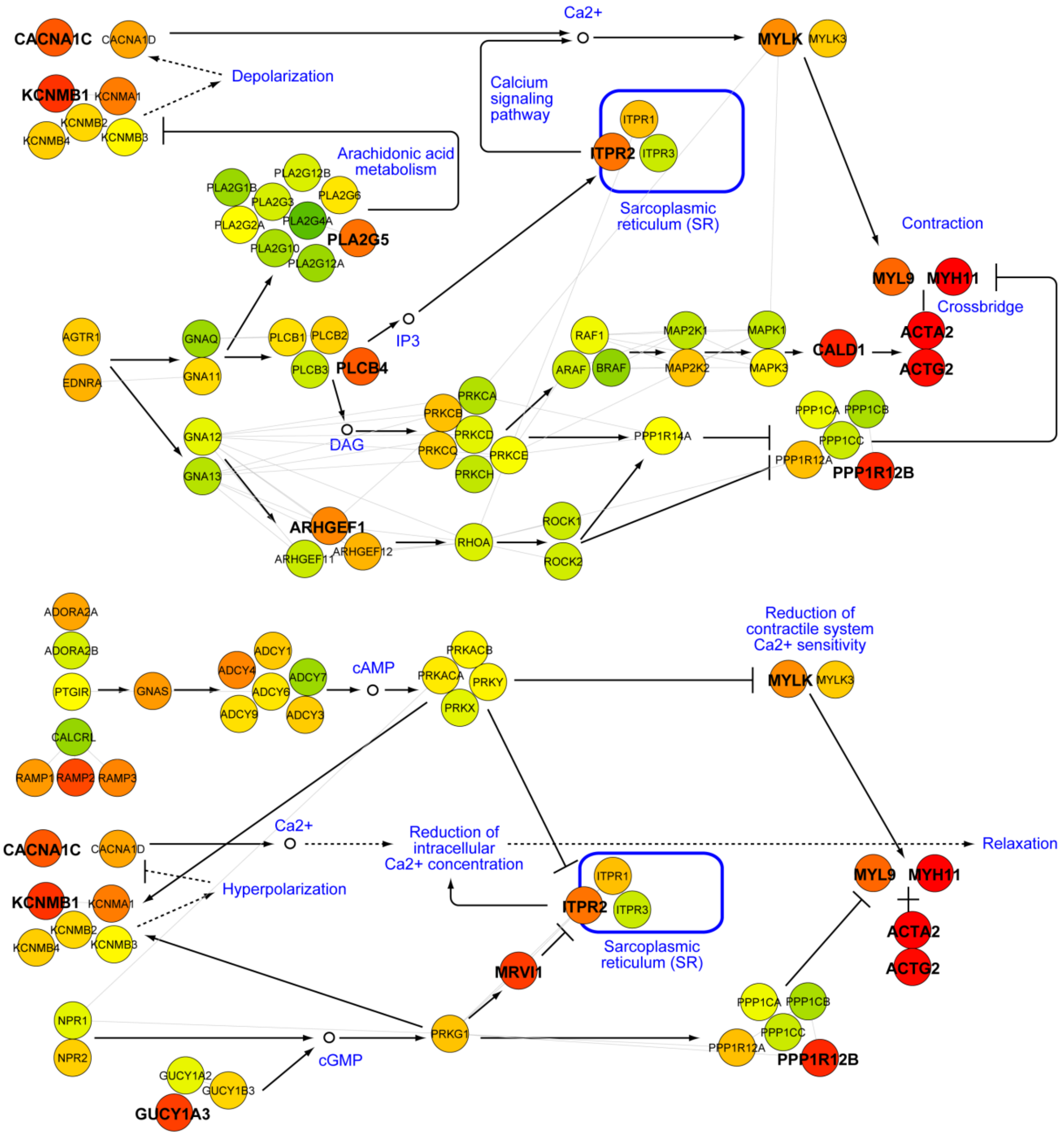

(E) Presumptive targeting interactions between differentially expressed miRNA and mRNA in the focal adhesion pathway.

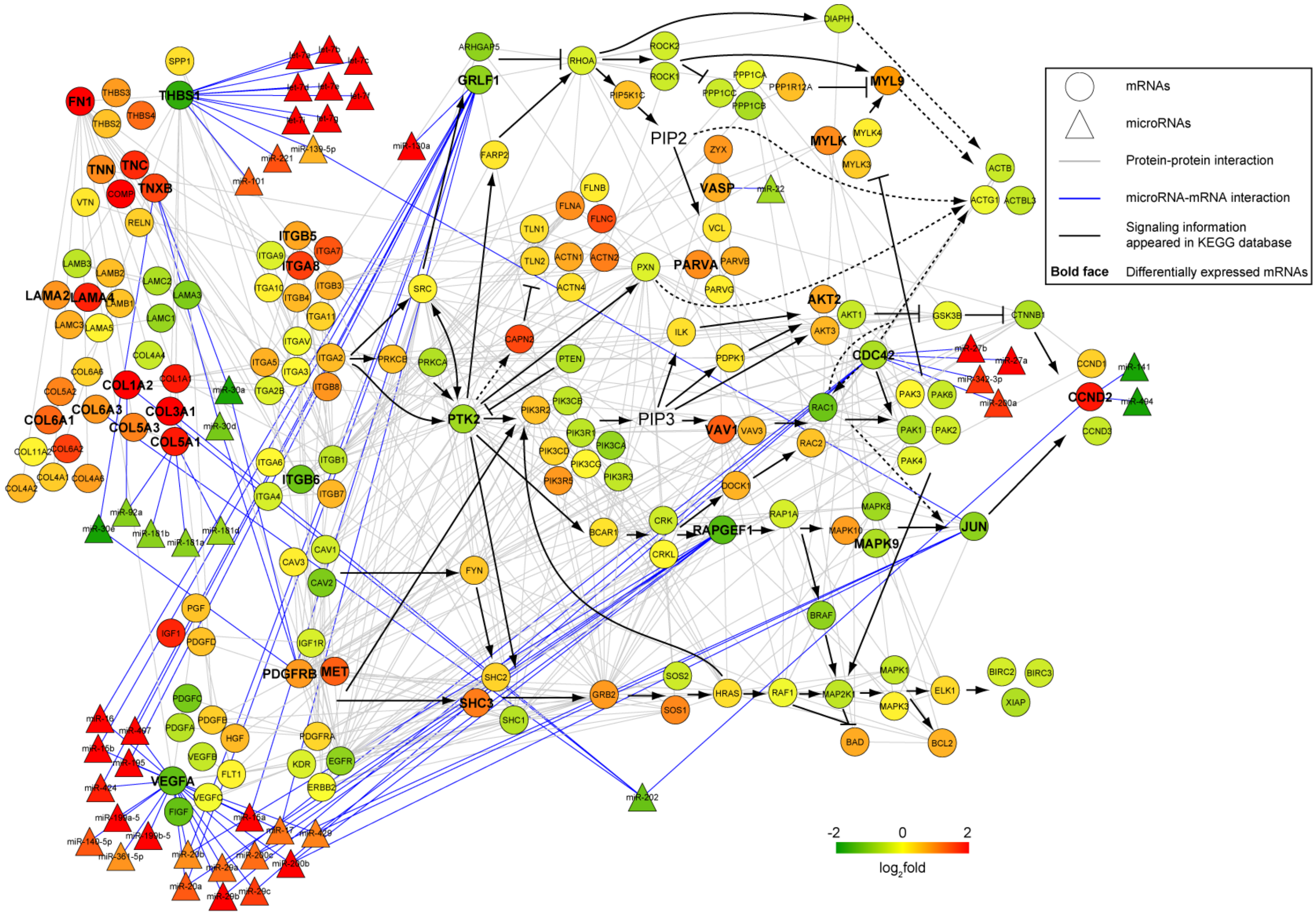

Supplement: Additional file 6 — Key pathways involved in the ILDs. [file 1755-8794-4-8-S6.PDF]
