# Supplementary material for: Systems biology of interstitial lung diseases: integration of mRNA and microRNA expression changes
Source: BMC Med Genomics. 2011 Jan 17;4:8. doi: 10.1186/1755-8794-4-8 (PMC3035594; doi:10.1186/1755-8794-4-8)

Additional file 7. Modules of the transcription factor and miRNA mediated-network based on KEGG pathway representation. DEGs that mapped to similar pathways were grouped together. Five modules were defined that represented a hierarchy of functional pathways: metabolism, cellular processes, environmental information processing, genetic information processing and human disease. DEGs belonging to multiple modules (e.g. *PLCB4* is involved in 14 KEGG pathways related to metabolism, cellular processes, environmental information processing and human disease) were grouped into a single module and placed at the center of the network (yellow highlight).

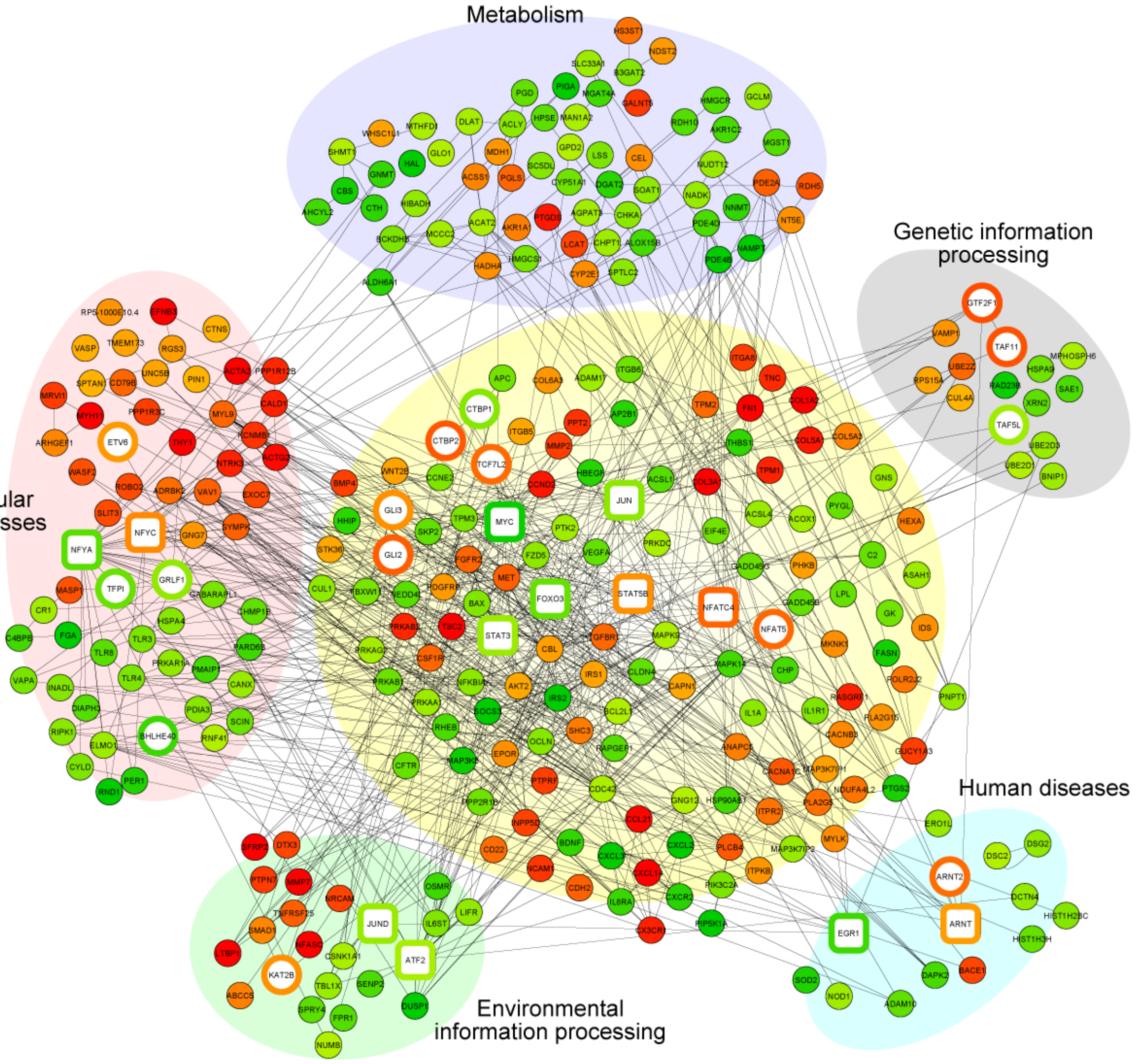

Supplement: Additional file 7 — Modules of DEGs based on KEGG pathways. [file 1755-8794-4-8-S7.PDF]
