# Supplementary material for: Systems biology of interstitial lung diseases: integration of mRNA and microRNA expression changes
Source: BMC Med Genomics. 2011 Jan 17;4:8. doi: 10.1186/1755-8794-4-8 (PMC3035594; doi:10.1186/1755-8794-4-8)

**Additional file 8**. ZEB1 binding sites upstream of the miR-23a cluster were identified using tfbsConsSites table from UCSC database (Hg18 assembly). Two putative ZEB1 binding sites, shown as blue lines in the figure, were found around 5kb and 7kb upstream of miR-23a cluster, respectively. The first one is indicated by TRANSFAC position weight matrix, V$AREB6_03, represents "VNRCACCTGKNC" motif and the other one, V$AREB6_01 does "NNYNYACCTGWVT" motif. Both of them contain E-box motif, "CANNTG".

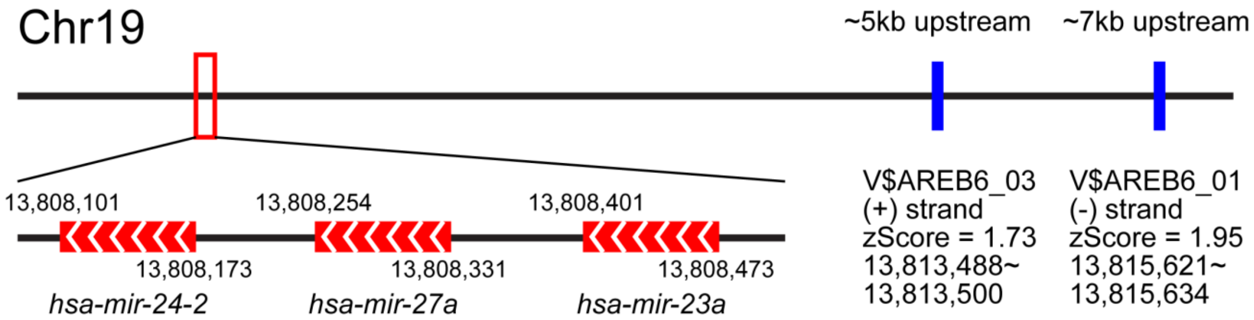

Supplement: Additional file 8 — Location of Zeb1 binding sites in the miR-23 distal promoter. [file 1755-8794-4-8-S8.PDF]
